# High-resolution genome-wide association study of a large Czech collection of sweet cherry (*Prunus avium* L.) on fruit maturity and quality traits

**DOI:** 10.1093/hr/uhac233

**Published:** 2022-10-19

**Authors:** Kateřina Holušová, Jana Čmejlová, Pavol Suran, Radek Čmejla, Jiří Sedlák, Lubor Zelený, Jan Bartoš

**Affiliations:** Institute of Experimental Botany of the Czech Academy of Sciences, Centre of the Region Haná for Biotechnological and Agricultural Research, Šlechtitelů 31, Olomouc, 779 00, Czech Republic; Research and Breeding Institute of Pomology Holovousy Ltd., Holovousy 129, Holovousy, 508 01, Czech Republic; Research and Breeding Institute of Pomology Holovousy Ltd., Holovousy 129, Holovousy, 508 01, Czech Republic; Research and Breeding Institute of Pomology Holovousy Ltd., Holovousy 129, Holovousy, 508 01, Czech Republic; Research and Breeding Institute of Pomology Holovousy Ltd., Holovousy 129, Holovousy, 508 01, Czech Republic; Research and Breeding Institute of Pomology Holovousy Ltd., Holovousy 129, Holovousy, 508 01, Czech Republic; Institute of Experimental Botany of the Czech Academy of Sciences, Centre of the Region Haná for Biotechnological and Agricultural Research, Šlechtitelů 31, Olomouc, 779 00, Czech Republic

## Abstract

In sweet cherry (*Prunus avium* L.), quantitative trait loci have been identified for fruit maturity, colour, firmness, and size to develop markers for marker-assisted selection. However, resolution is usually too low in those analyses to directly target candidate genes, and some associations are missed. In contrast, genome-wide association studies are performed on broad collections of accessions, and assemblies of reference sequences from Tieton and Satonishiki cultivars enable identification of single nucleotide polymorphisms after whole-genome sequencing, providing high marker density. Two hundred and thirty-five sweet cherry accessions were sequenced and phenotyped for harvest time and fruit colour, firmness, and size. Genome-wide association studies were used to identify single nucleotide polymorphisms associated with each trait, which were verified in breeding material consisting of 64 additional accessions. A total of 1 767 106 single nucleotide polymorphisms were identified. At that density, significant single nucleotide polymorphisms could be linked to co-inherited haplotype blocks (median size ~10 kb). Thus, markers were tightly associated with respective phenotypes, and individual allelic combinations of particular single nucleotide polymorphisms provided links to distinct phenotypes. In addition, yellow-fruit accessions were sequenced, and a ~ 90-kb-deletion on chromosome 3 that included five MYB10 transcription factors was associated with the phenotype. Overall, the study confirmed numerous quantitative trait loci from bi-parental populations using high-diversity accession populations, identified novel associations, and genome-wide association studies reduced the size of trait-associated loci from megabases to kilobases and to a few candidate genes per locus. Thus, a framework is provided to develop molecular markers and evaluate and characterize genes underlying important agronomic traits.

## Introduction

Sweet cherry (*Prunus avium* L.) is a vegetatively propagated perennial crop grown for its tasty fruits. It is a diploid species in the Rosaceae with a relatively small genome of approximately 340 Mb (2n = 16) [1]. The first genome sequenced was that of the Japanese leading cultivar Satonishiki in 2017 [1], and then, that of Tieton, the most popular cherry cultivar in China, was sequenced and annotated [2]. Sweet cherries are popular worldwide but are not available for several months because of a short harvest season and a limited shelf life (7 to 10 days) [3]. To improve sweet cherry cultivars, breeding programs focus primarily on extending the harvest season and increasing fruit size, quality and attractiveness [4, 5].

It is a challenge to breed new sweet cherry cultivars because of relatively small numbers of progeny compared to number of pollinated flowers. For example, Quero Garcia [6] reported data from eleven seasons when 266 115 pollinated flowers produced 12 488 fruits (4.1%) and only 2625 (0.99%) hybrids were obtained under germination rate of 21%. Complication for breeding is also a long juvenile period and many labour-intensive processes needed and time from initial cross to new genotype commercial availability is often over 20 years. Because traditional breeding is expensive and time-consuming, any strategy that accelerates the process and improves its efficiency has high potential economic impact [7]. The most promising approaches use molecular markers associated with phenotypical and phenological traits in marker-assisted selection (MAS). Estimates are that MAS saves ~$80 000 per year in resources when selecting 3000 to 3500 sweet cherry seedlings [8]. Nevertheless, only a few molecular markers and candidate genes have been identified and used in breeding (see examples below; for review see Quero Garcia [9]), and strategies to develop new markers have been limited primarily to traditional mapping and quantitative trait locus (QTL) analysis.

Many breeders aim to prolong the ripening period by identifying molecular markers associated with maturation and harvest times. Such QTLs were mapped recently on several regions on chromosome 4 for sweet cherry [10–13] and also for other *Prunus* species, including peach [14], apricot [15], and Japanese plum [16] and the NAC transcription factors have been proposed as candidate genes [12, 13]. Additional QTLs have also been identified on chromosomes 1, 2, 3, and 5 [13].

To customize fruit colour according to consumer preferences, Sooriyapathirana et al. [17] identified major QTLs for fruit and flesh colours on chromosome 3 with the candidate gene *PavMYB10*, as well as less significant QTLs on chromosomes 6 and 8. According to Lin-Wang et al. [18], the same gene was associated with regulation of the anthocyanin biosynthetic pathway, and not only in sweet cherry. Overlapping QTLs for fruit skin and flesh colours have also been identified on chromosome 3 [19]. In addition, *PavMYB10.1* gene polymorphism has been proposed as causative for fruit colour [20]. Three alleles of *PavMYB10.1* affect fruit colour: a wild-type allele associated with red; an allele with a deletion that leads to a premature stop codon associated with marbled appearance; and an allele with an uncharacterized deletion associated with yellow [20].

Fruit firmness influences resilience to bruise damage and shelf life, and loci associated with firmness are on all chromosomes [11, 13, 21–23], with hot spots on chromosomes 1, 4, and 6. Candidate genes are associated with expansins, plant cell wall-modifying enzymes, and hormone signalling pathways involved in fruit maturation and ripening [22].

Fruit weight and size affect producer profits, because larger fruits are more valued and easily harvested. Therefore, molecular markers for fruit size were among the first used in MAS in sweet cherry. The QTLs associated with fruit size and weight are on chromosomes 1, 2, 3, 5, and 6 [13, 21, 23–25]. Simple sequence repeat (SSR) markers CPSCT038 and BPPCT034 from chromosome 2 linked with fruit size and weight by Zhang et al. [24] and Rosyara et al. [25] have been the most frequently used. Notably, the two SSR markers are close to the candidate gene *PavCNR12* for fruit size described by De Franceschi et al. [26].

Traditional genetic mapping was initially used to identify QTLs with SSRs and cleaved amplified polymorphic sequences (CAPS) as molecular markers [10, 11, 17, 24]. Recently, universal 6 K [8] and 6 + 9 K [27] single nucleotide polymorphism (SNP) arrays have been used to map QTLs of sweet and sour cherries [13, 19, 21–23]. Nevertheless, those approaches provide limited resolution in the gene-rich cherry genome. For example, although *PavCNR12* is a promising candidate gene for fruit weight, a huge distance remains between the most significant marker G2SSR1576 associated with the respective QTL and candidate gene [26]. In the reference genome of Tieton v2.0 [2], the two are separated by approximately 210 kb and more than 20 genes. Although both SNP arrays have greatly increased marker density, such progress is apparently insufficient to link traits to a few candidate genes. Sweet cherry has only 3955 reliable polymorphic SNPs in the 6 + 9 K SNP array, for an average density of one SNP per 86 kbp of genome. Moreover, SNPs anchored on the arrays are not distributed evenly, and each chromosome has at least one SNP-free gap larger than 500 kb [27]. Although the marker density is sufficient for QTL analysis, it limits identification of candidate genes associated with particular traits.

Whole-genome sequencing provides unsurpassed density of SNP markers, in contrast to other approaches. Nearly 2 million SNPs were recently identified among 21 sweet cherry accessions [28]. Furthermore, linkage disequilibrium (LD) between SNPs decays to 50% of its maximum at 5 kb and to 90% at 55 kb [28]. Such rapid decay of LD suggests that genome-wide association studies (GWAS) can provide high-resolution mapping of genes associated with agriculturally significant sweet cherry traits and decrease the possibility of missed associations.

In this study, GWASs were used to exploit the genetic variation in a collection of sweet cherry accessions and address the following objectives: i) identify SNP markers significantly associated with the most desirable sweet cherry traits of harvest time and fruit colour, firmness, and size; ii) use the SNP markers to validate locations of known QTLs and vice versa; iii) advance from analysis of QTLs to identification of candidate genes.

## Results

### Genotyping and genetic structure of population

**Figure 1:**
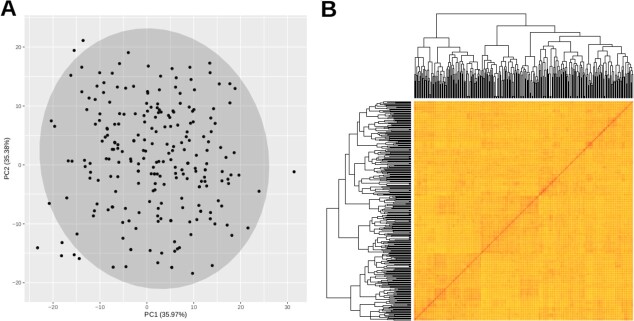
**Collection of Genetic Resources population structure analysis. A)** Principal component (PC) analysis plot of the first two PCs identified from 235 accessions based on 1 767 106 single nucleotide polymorphisms. **B)** Heat map of a kinship matrix estimated using the VanRaden algorithm. Both plots were generated with GAPIT.

Paired-end sequencing on Illumina NovaSeq 6000 yielded a 4.15-Tb sequence. Individual samples contributed 7.6–36.8 Gb (BioProject in SRA archive: PRJNA813711) representing 22.4–108.1X genome coverage, assuming a sweet cherry genome of 340 Mb [2]. The SNP calling revealed 1 767 106 high-quality SNPs (average of one SNP per 192 bp) in all accessions. The SNPs were evenly distributed among accessions ranging from 1 162 493 in SB135 to 1 846 267 in SB033. Principal component zero showed the highest value of Bayesian information criterion (BIC) in principal component analysis (PCA) performed with the Collection of Genetic Resources (CGR). Therefore no principal component controlled the population structure, and no subpopulations were revealed (Fig. 1). There was also no strong sample clustering in the kinship matrix.

### Phenotyping and genome-wide association study

Phenotyping revealed broad ranges of values for phenotypical and phenological characteristics from 2012 to 2020, including *time of harvest*, *fruit colour*, *flesh colour*, *firmness of skin*, *firmness of flesh*, *value of penetration*, *fruit bruiseness*, *fruit length*, *fruit width*, *fruit thickness*, and *weight of fruit* (Table 1; Fig. S1). Generally, traits in the CGR accessions were more variable than those in breeding materials (BM) accessions (Table 1; Fig. S1). There was an apparent shift in “colour characteristics” between the two populations. The CGR included accessions with fruit and flesh colours ranging from yellow/white to dark red. In BM accessions, yellow fruits were absent. Other phenotypic differences reflected breeding goals, with larger fruits and better firmness characteristics usually associated with later ripening. Average “size characteristics” were larger in BM than in CGR accessions; for example, average *weight of fruit:* 5.97 g in CGR vs. 8.76 g in BM. *Firmness of flesh* and *fruit bruiseness* values were also lower in CGR accessions, whereas *firmness of skin* values were higher. *Time of harvest* ranged from 0 to 50.63 and from 8.63 to 54.6 days (after reference SA993) in CGR and BM accessions, respectively, indicating later ripening in BM accessions.

**Table 1:** Statistical evaluation of traits concerning time of harvest and fruit colour, size, weight, and firmness

**Characteristic**	**Collection of genetic resources**					**Breeding materials**				
	**Average**	**Std.Dev**	**Median**	**Min**	**Max**	**Average**	**Std.Dev**	**Median**	**Min**	**Max**
**Time of harvest** ^**a**^	23.87	8.60	25.67	0.00	50.63	29.95	9.72	31.14	8.63	54.60
**Fruit colour** ^**b**^	7.16	1.91	7.89	1.00	9.00	7.19	0.42	7.13	6.13	8.17
**Flesh colour** ^**b**^	6.30	2.14	7.00	1.00	9.00	5.98	0.73	6.00	4.00	8.00
**Firmness of skin** ^**b**^	6.78	0.90	7.00	3.71	8.25	6.37	0.80	6.50	4.00	7.67
**Fruit flesh firmness** ^**b**^	6.11	1.23	6.38	2.43	8.67	6.44	1.18	6.75	3.33	8.00
**Fruit bruiseness** ^**b**^	6.62	1.10	6.86	2.29	8.80	7.03	0.96	7.00	4.33	9.00
**Value of penetration** ^**c**^	0.40	0.09	0.38	0.16	0.78	58.37	8.14	57.17	41.06	78.15
**Fruit length** ^**d**^	21.31	1.82	21.30	16.70	26.98	23.50	1.50	23.67	18.70	26.04
**Fruit width** ^**d**^	22.62	2.02	22.53	16.70	28.05	25.83	1.38	26.04	22.33	29.24
**Fruit thickness** ^**d**^	19.84	1.72	19.83	12.02	24.67	22.12	1.36	22.14	18.27	25.10
**Weight of fruit** ^e^	5.97	1.31	5.92	2.88	10.98	8.76	1.24	8.98	5.86	11.23

a Days after reference cultivar SA993.

b Values based on 9-point classification scale.

c Collection of Genetic Resources: evaluated in years 2015 to 2020 only, (kg.cm^−2^); Breeding Materials: index of firmness, 1 to 100.

d Millimetres.

e Grams.

Before final GWASs, performances of different statistical models were compared for all combinations of phenotype characteristics and models (for details, see Materials and Methods). For most traits, the FarmCPU model provided values of the genomic inflation factor (lambda) closest to 1 (Table S1) and hence was selected. The GWASs revealed 59 SNPs associated with eleven traits (see below; Tables S2–S5).

### Time of harvest

**Figure 2:**
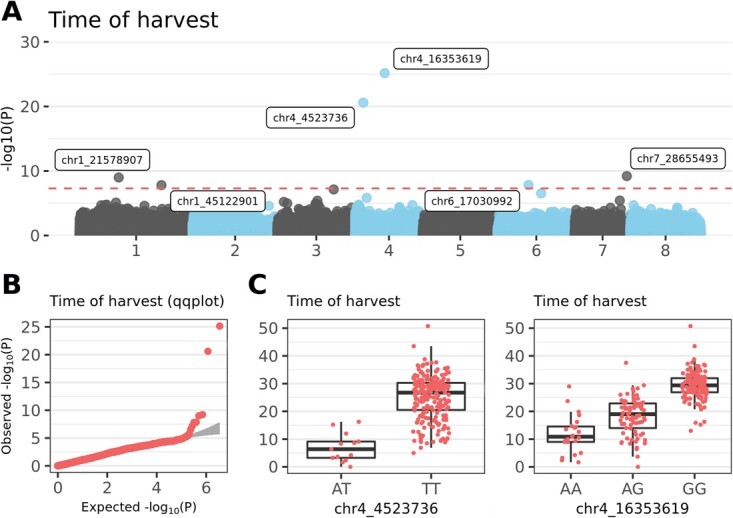
**Genome-wide association study (GWAS) for *time of harvest*. A)** Manhattan plot, with red dashed line indicating Bonferroni’s threshold. **B)** Quantile–quantile (qq) plot for the GWAS on *time of harvest* phenotypes. **C)** Allele-effect box plots for SNP markers with the highest association with *time of harvest*. Phenotypical value is number of days after the reference cultivar Kisinevskaja (SA993).

In the GWAS for *time of harvest*, five associated markers were identified (Table S2). The strongest association was found on chromosome 4 at positions 16 353 619 and 4 523 736 bp (Fig. 2; Fig. S2). Homozygous genotype AA for SNP marker **chr4_16 353 619** (A/G, *p*-value 7.21e^−26^) was significantly linked with accessions with early ripening, whereas the allele combination GG was typical for late-ripening samples. Cultivars with genotypes AA, AG, and GG had average *time of harvest* of 11.9, 18.4, and 29.4 d, respectively after the reference very-early ripening cultivar Kisinevskaja (SA993). For SNP marker **chr4_4 523 736** (A/T, *p*-value 2.57e^−21^), only heterozygotes and the homozygous TT combination were detected in CGR accessions. Whereas heterozygous genotypes characterized early-ripening accessions; TT alleles indicated significantly later ripening (7.0 vs. 25.0 d, after reference cultivar SA993). In contrast, heterozygous genotypes TC for SNP marker **chr1_21578907** (T/C, *p*-value 1.03e^−9^) are associated with later ripening compared to accessions with homozygous CC genotype (36.7 vs. 23.6 d, after reference cultivar SA993).

### Colour characteristics

**Figure 3:**
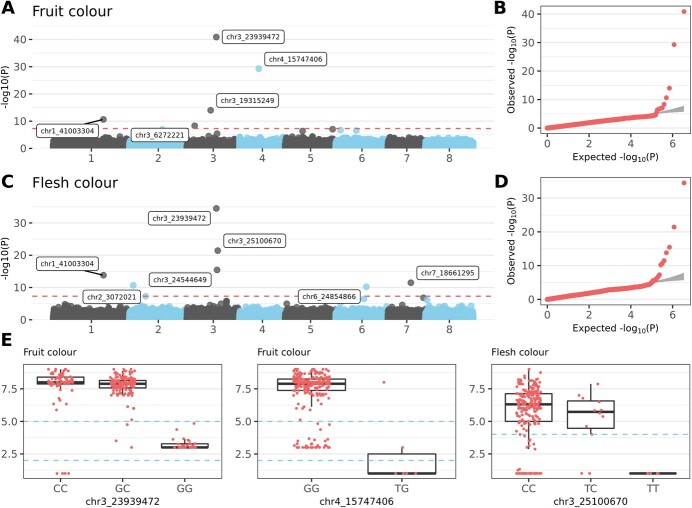
**Genome-wide association study (GWAS) for colour characteristics. A)** Manhattan plot based on FarmCPU model for *fruit colour* trait. Red dashed line indicates Bonferroni’s threshold. **B)** Quantile–quantile plot for the GWAS on *fruit colour* phenotypes. **C)** Manhattan plot for *flesh colour* trait. Red dashed line indicated Bonferroni’s threshold. **D)** Quantile–quantile plot for the GWAS on *flesh colour* phenotypes. **E)** Allele-effect box plots for SNP markers. Phenotypical values between two blue dashed lines represent accessions with marble *fruit colour*, with lower and higher values corresponding to yellow and red fruits, respectively. The blue dashed line in *flesh colour* divides accessions with yellow (under line) and red phenotypes.

Ten SNP markers were significantly associated (above Bonferroni’s threshold) with the two interconnected phenotype characteristics *fruit colour* and *flesh colour* (Table S3). Individual phenotypes were highly correlated (*r* > 0.92; Fig. S3). *Fruit colour* varied from yellow to dark red, but only four accessions had yellow skin. Nevertheless, several SNPs on chromosomes 3 and 4 were strongly associated with the trait. The most significant SNP **chr3_23939472** (G/C) was identified with a *p*-value of 1.37e^−41^. Accessions with marble coloured fruit skin possessed primarily GG alleles for the particular SNP. By contrast, the CC allele combination characterized either dark red or yellow skin phenotypes (Fig. 3; Fig. S4). An SNP for *fruit colour* identified on chromosome 4 (**chr4_15747406**; T/G) was also significant with a *p*-value of 5.31e^−30^. However, the minor allele frequency for that SNP was only 0.01, with most accessions homozygous with alleles GG. All four yellow-coloured fruit accessions were heterozygous (GT) for the locus.

The most significant marker associated with *flesh colour* was identified on chromosome 3. Homozygous genotype GG for SNP marker **chr3_23939472** (G/C, *p*-value 3.15e^−35^) indicated yellow flesh in those particular accessions. Homozygous genotype CC for the same SNP was associated with darker phenotypes than those of heterozygous genotype CG. Notably, two SNPs (chr3_23939472 and chr1_41003304) were associated with both *fruit colour* and *flesh colour*.

### Deletion in MYB10 region

**Figure 4:**
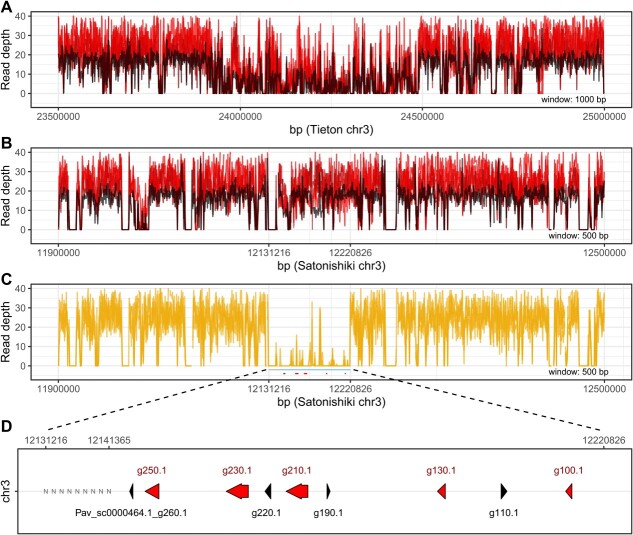
**Identification of deletion in MYB10 region. A)** Read depth (window 1 kb) of four accessions with red-coloured fruits (red lines) and Tieton variety also with red-coloured fruits (black line). Reads were mapped to the region with MYB factors on chromosome 3 in the Tieton reference genome. **B)** Read depth (window 500 bp) of the same four accessions (red lines) and Tieton (black line) mapped to the Satonishiki reference genome revealed after mapping to the region with MYB10 transcription factors on chromosome 3 (corresponding to homologous region in the Tieton genome in **A**). **C)** Read depth (window 500 bp) of four accessions with yellow-coloured fruits (yellow lines) mapped to the Satonishiki reference genome (as for red accessions in **B**). Blue line highlights the deletion found in accessions with yellow-coloured fruits, and red lines mark MYB genes. **D)** Zoom of deletion found in accessions with yellow-coloured cherries with MYB genes (red arrows) annotated in the Satonishiki reference genome. At the beginning of deleted region, the sequence was not defined in the reference (NNN); hence, it was not possible to accurately determine the size of the deletion.

In closer examination of SNPs associated with *fruit colour*, a cluster of MYB transcription factors on chromosome 3 was revealed in the reference Tieton genome between SNP markers chr3_23939472 and chr3_24544649. The region is known contain *MYB10.1*, which is involved in determination of fruit colour; with yellow fruits in accessions with homozygous deletion in the region [20]. To determine size of the deletion, reads mapped in the region were compared in accessions with yellow and red fruits. Notably, although mapped to the Tieton reference sequence, coverage calculated for red cherry accessions (including Tieton) decreased to half in the region containing MYB genes (compared with the surrounding region; Fig. 4A). This finding indicated that region was duplicated in the Tieton reference genome. By contrast, in all accessions with red *fruit colour* (including Tieton), the region of MYB genes was covered evenly with the same coverage as surrounding regions when mapped to the Satonishiki reference genome [1] (Fig. 4B). To summarize, it was concluded that duplication in the Tieton reference genome was an artificial product of the assembly process.

When reads for accessions with yellow *fruit colour* were mapped to the Satonishiki reference genome, a significant decrease in coverage (nearly to zero) was identified in a region ranging from 12.131 to 12.220 Mb on chromosome 3 (Fig. 4C). The deletion was approximately 90 kb in length and contained a region with five MYB transcription factors (Fig. 4D). According to pedigree analysis of yellow-fruited cherries using SSR markers, SB083 (Stark gold sweet cherry) and SB124 (Dönissenova) were likely the same genotype as these two varieties had identical alleles for each analysed SSR marker. Moreover, they shared one of the two alleles for each SSR marker with another yellow-fruited accession SB377 (Droganova). In contrast, SB134 (41/2 Ljana × Cherry self fert. 4.6) had a different origin, and another accession in the CGR, SB077 (Cherry self fert. 4.6) with dark red fruits, was identified as its possible parent. Accession SB077 had a heterozygous deletion in the same region as that in yellow-fruited cherries (Fig. S5).

### Firmness

**Figure 5:**
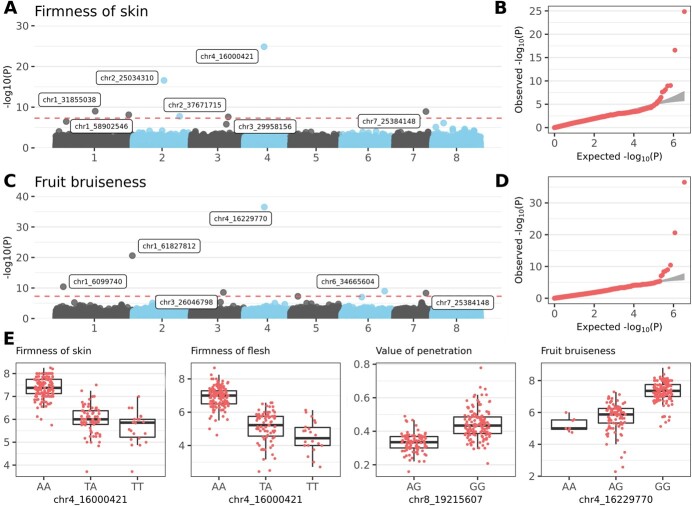
**Genome-wide association study (GWAS) for firmness characteristics. A)** Manhattan plot for *firmness of skin* trait. Red dashed line indicates Bonferroni’s threshold. **B)** Quantile–quantile plot for the GWAS on *firmness of skin* phenotypes. **C)** Manhattan plot for *fruit bruiseness* trait. Red dashed line indicates Bonferroni’s threshold. **D)** Quantile–quantile plot for the GWAS on *fruit bruiseness* phenotypes. **E)** Allele-effect box plots for SNP markers with the highest association with *firmness of skin*, *firmness of flesh*, *value of penetration*, and *fruit bruiseness* phenotypes. Phenotype values for *firmness of skin*, *firmness of flesh*, and *fruit bruiseness* are based on a 9-point classification scale, and units for *value of penetration* are kg.cm^−2^.

Eighteen SNP markers (Table S4) were associated with the phenotypes *firmness of skin*, *firmness of flesh*, *value of penetration* and *fruit bruiseness*, which were also correlated (Fig. S6). The strongest association was found for markers on chromosome 4 around 16 Mbp. *Firmness of skin* and *firmness of flesh* were significantly associated with marker **chr4_16000421** (T/A, *p*-values 1.41e^−25^ and 1.68e^−25^, respectively). Accessions with homozygous genotype AA had significantly stronger *firmness of skin* and *firmness of flesh* than those with TA and TT allele combinations (Fig. 5; Fig. S7). For SNP marker **chr8_19215607** (A/G *p*-value 6.92e^−17^), homozygous genotype GG was associated with higher *value of penetration* than that of the AG genotype. Accessions with homozygous genotype GG for SNP marker **chr4_16229770** (A/G, *p*-value 3.06e^−37^) had reduced *fruit bruiseness*, as indicated by higher values on a nine-point classification scale than those of accessions with allele combinations AA and AG (Fig. 5). By contrast, homozygous genotypes CC and AA for markers **chr1_61827812** (C/A, *p*-value 2.55e^−21^) and **chr1_6099740** (A/G, *p*-value 3.81e^−11^), respectively, indicated relatively high bruiseness. In addition, marker chr7_25384148 was associated with *fruit bruiseness* as well as *firmness of skin*.

### Size and weight of fruit

**Figure 6:**
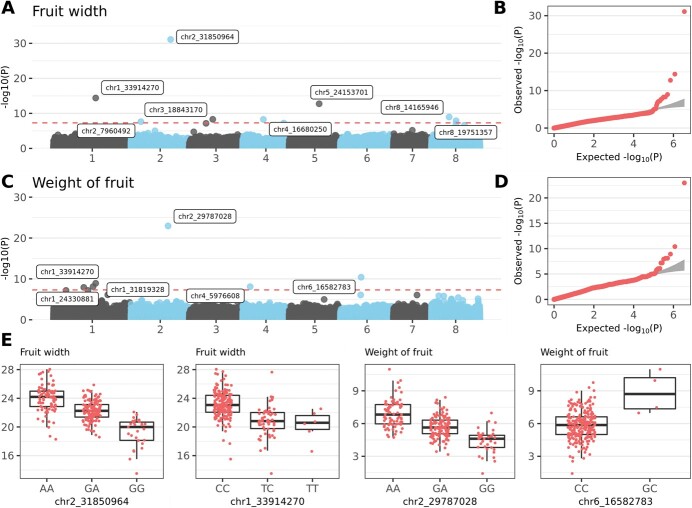
**Genome-wide association study (GWAS) for size characteristics. A)** Manhattan plot for *fruit width* trait. Red dashed line indicates Bonferroni’s threshold. **B)** Quantile–quantile plot for the GWAS on *fruit width* phenotypes. **C)** Manhattan plot for *weight of fruit* trait. Red dashed line indicates Bonferroni’s threshold. **D)** Quantile–quantile plot for the GWAS on *weight of fruit* phenotypes*.***E)** Allele-effect box plots for SNP markers with the highest association with *fruit width* and *weight of fruit* phenotypes. Phenotypical values were measured in millimetres for *fruit width* and in grams for *weight of fruit*.

Twenty-six SNP markers were associated with *fruit length*, *fruit width*, *fruit thickness* and *weight of fruit* (Table S5), which were also highly correlated (Fig. S8). Markers positioned around 29 Mb on chromosome 2 had the strongest associations. Markers associated with *fruit length* were localized on six chromosomes. Accessions with relatively short length were characterized, for example, by homozygous allele combinations TT, GG, TT, and GG for SNP markers **chr2_29776624 (**T/C, *p*-value 2.88e^−16^), **chr6_34823266 (**G/T, *p*-value 1.28e^−11^), **chr8_27088406 (**T/A, *p*-value 1.04e^−10^), and **chr4_5976608 (**G/C, *p*-value 2.35e^−09^), respectively (Fig. S9). One SNP marker **chr2_31850964** (G/A, *p*-value 7.94e^−32^) (of eight total) was very strongly associated with *fruit width* (Fig. 6). Accessions with homozygous genotype GG had smaller fruit widths than those of accessions with homozygous genotype AA, with approximately 4 mm the average difference. For *fruit thickness*, the strongest association with *p*-value 2.53e^−13^ was also found on chromosome 2. Homozygous genotypes GG at locus **chr2_29776725** (G/A) had smaller fruit thickness than GA heterozygotes and AA homozygotes, with ~2 and ~ 3.5 mm thedifference, respectively. For *weight of fruit*, associated SNPs were identified, for example, on chromosomes 2 and 6. Accessions with homozygous genotypes GG for marker **chr2_29787028** (G/A, *p*-value 1.11e^−23^) and CC for marker **chr6_16582783** (G/C, *p*-value 3.94e^−11^) had lower weights than those of other variants (Fig. 6).

**Figure 7:**
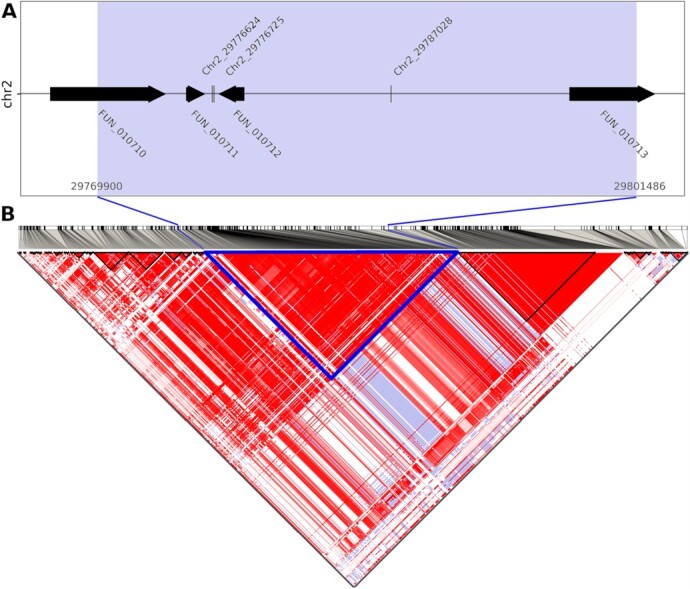
**Detailed view of genomic region identified by genome-wide association study for size traits. A)** Single nucleotide polymorphisms associated with *fruit length* (chr2_29776624), *fruit thickness* (chr2_29776725), and *weight of fruit* (chr2_29787028) and four genes annotated in the Tieton reference genome found within one haplotype block (highlighted in blue). **B)** Linkage disequilibrium (LD) analysis of the particular genomic region. The triangles indicate individual haplotype blocks. Two hundred and ninety SNP markers cosegregated with particular traits (blue haplotype block). The strength of LD between individual SNP pairs is displayed using a colour code, with strong LD in red and weak LD in white.

Two markers were associated with two different traits: chr1_33914270 with *fruit width* and *weight of fruit* and chr4_5976608 with *fruit length* and *weight of fruit*. In addition, SNPs chr2_29776624, chr2_29776725 and chr_29787028 associated with *fruit length*, *fruit thickness* and *weight of fruit*, respectively colocalized in a single haplotype block (see below, Fig. 7).

### Identification of haplotype blocks and candidate genes

Haplotype blocks co-inherited in the CGR population were successfully assigned for 46 significant SNPs (78%) (Supplementary Data). Blocks ranged from 2 to 307 SNPs and from 10 bp to 58.6 kb in size. Medial size of assigned haplotype blocks was 10.1 kb. Thirteen remaining SNPs were not part of any co-inherited SNP group. Nine SNPs directly hit the region of candidate genes between start and stop codon. Number of candidate genes in individual haplotype blocks ranged from one to nine. A total of 141 candidate genes were identified, and homology-based annotation indicated function for 104 (Supplementary Data; for more information on possible functional significance of candidate genes, see the Discussion). One of the haplotype blocks (290 SNPs; ~31.6 kb) included three different SNPs associated with three different traits, all linked to fruit size (Fig. 7). Four genes were inherited within this block, including strong candidates for determination of fruit size (for more details, see Discussion).

### Verification of genome-wide association study results

The GWAS results were verified using a second set of 64 additional accessions (denoted BM), which were processed in the same way as CGR accessions up to SNP calling. In a PCA of all 299 samples (CGR + BM), subpopulations were not identified, but BM accessions were slightly shifted compared with CGR accessions (Fig. S10). Most SNP markers had similar allele effects on phenotypes in both sets (Figs. S2, S4, S7, S9), although there were several notable but expected observations.

For *time of harvest*, the difference in minimal values between BM and CGR was 8.63 d. The phenotypic values suggested loss of allele combinations for early ripening in BM accessions. Accordingly, no BM accession had the AT allele combination for marker chr4_4 523 736, which was associated with early time of harvest (Fig. S2). For colour phenotypes, allele combinations GG and TT in BM accessions for markers chr3_23939472 and chr3_25100670, respectively, were absent (Fig. S4). Because those alleles are typical for fruits with non-red skin and flesh, losses were likely because the goal was to breed primarily red-coloured fruits. For *fruit bruiseness*, the AA allele combination of SNP markers chr4_16229770 and chr1_6099740 was consistently missing in the BM population because of selection for less bruiseable fruits (Fig. S7). For *weight of fruit*, allele combinations GG, TT, and GG were absent in BM accessions for markers chr2_29787028, chr1_33914270, and chr4_5976608, respectively (Fig. S9). This result was consistent with selection toward bigger fruits in BM, because homozygous GG and TT genotypes characterized CGR accessions with the smallest fruits.

Relevance of SNPs was also confirmed by comparing genomic positions with previously known QTLs for particular traits. Twenty-two of the SNPs colocalized with known QTLs (Tables S2–S5), and especially, all SNPs most significant for individual traits (except *fruit length* and *value of penetration*) were associated with loci previously described as QTLs for those particular or similar traits (i.e. published QTLs for fruit size were universally used for all analysed dimensions). Furthermore, three (of six total) SNPs associated with two different but highly correlated traits were associated with published QTLs. Additionally, SNPs associated with *fruit colour* marked unambiguously deletion of a region containing five MYB transcription factor genes, with *MYB10.1* responsible for differences in sweet cherry skin colour [20].

## Discussion

Molecular markers for valuable sweet cherry traits are rare and thus need to be developed and validated. Previously, the most precise approaches to identify QTLs associated with important traits used the cherry 6 + 9 K SNP array [19, 27], with approximately one SNP per 86 kbp of genome. With the whole-genome sequencing used in this study, resolution was refined to one SNP per ~200 bp. In the GWASs at such a high density of markers, haplotype blocks co-inherited in the population could be identified, and the analysis could shift from identification of QTLs to a few candidate genes (for discussion of the most relevant candidate genes, see below). This progress in quantitative trait analysis offers opportunities to explore allelic variation of specific genes in a wider collection of accessions (including sweet cherry relatives) and access new alleles for breeding. In the current analysis, several significant SNPs showed low minor-allele frequency (<0.05), and consequently, particular homozygous combinations of alleles were missing in the CGR accessions. Notably, heterozygous allele combinations of those SNPs provided significant phenotype shifts compared with major-allele homozygotes. For example, minor-allele homozygotes for chr4_4 523 736 and chr1_21578907 for *time of harvest* (if viable), could provide very early and late fruit ripening, respectively, and therefore extension of the harvest period.

The GWASs were performed using 235 accessions (CGR). The effects of the identified SNPs were then verified on anindependently evaluated set of 64 additional accessions (BM).Significance of markers was confirmed on trees with fundamentally different growing habits. While 20- to 30-year-old CGR trees were planted on vigorous mazzard rootstock, very young BM trees were grafted on semidwarf rootstock Gisela 5, with all expected consequences including those for tree height.Colocalization of most SNPs (including those with the highest significance) and known QTLs provided additional independentevidence of robustness of results. On the other hand, some candidate genes proposed in previous studies were missed by the GWAS in this study (for *time of harvest* NAC transcription factors [12, 13] and for *fruit weight PavCNR12* [26]). However,those candidate genes are localized in a vicinity of SNPs discovered in GWAS, although not in their haplotype blocks (see below). Three possible reasons might be responsible for the inconsistent results: 1) multiple neighbouring loci (genes) couldunderlie individual traits; 2) previously described candidates were falsely identified within broad QTL regions; 3) GWAS resultedin imprecise localization of associated SNPs (e.g. caused bymissing datapoints or sequence missassembly in particular region, which interrupt haplotype block). While candidate genes should be searched in some cases beyond particular haplotype blocks and further analysis will be needed to confirm their effect, GWAS undoubtedly highlighted trait-associated regions and provided SNP markers tightly linked to phenotypes for MAS.

### Time of harvest

The most significant SNPs identified by the GWAS were on chromosome 4, which is a hot spot for harvest and ripening traits according to previous studies [10, 12, 13]. The SNP with the lowest *p*-value, chr4_16 353 619, was mapped into the region of HD_Y4_56 [12], and chr4_4 523 736 was mapped into the qP-MD4.1^m^ region [13]. A significant SNP, chr1_21578907, was also positioned into qP-MD1.1^m^ [13]. Hence, three of five SNPs for *time of harvest* collocated with known QTLs.

Although NAC transcription factors (NAC5 and NAC25) are proposed as candidate genes [12, 13] based on associations with broad QTL regions, those genes did not overlap with the haplotype block identified for SNP marker chr4_16 353 619 in the current analysis (being ~300 kb apart). Instead, chr4_16 353 619 colocalized with the diacylglycerol kinase 5 gene (FUN_034033; gene nomenclature here and hereafter according to Wang et al. [2]). Diacylglycerol kinases are key signalling enzymes that phosphorylate diacylglycerol to phosphatidic acid (PA), a pivotal second messenger also potentially associated with fruit ripening [29]. Phosphatidic acid is triggered by many plant stimuli [30], including abscisic acid (ABA), the plant hormone that directs ripening in nonclimacteric sweet cherry [31]. Thus, PA can mediate ABA-controlled fruit ripening, indicating the association of diacylglycerol kinase with fruit maturity deserves further investigation.

The second most significant SNP for *time of harvest*, chr4_4 523 736, shared the haplotype block with four genes (FUN_032302 to FUN_032305), with the most interesting aquaporin *NIP1–1* (FUN_032302). Aquaporins form membrane water channels that are critical in controlling cell and tissue water content. Twenty-eight putative genes coding for aquaporins have been identified in the sweet cherry genome, and 16 are differentially expressed in developing fruits [32]. *NIP1–1* (named *PaNIP1;2a* by Chen et al. [32]) is expressed only in fruit skin, with the highest expression immediately preceding the phase of rapid fruit growth; however, its precise role in ripening remains unclear.

### Fruit colour

In the GWAS for *fruit colour* and *flesh colour*, the most significant SNPs were identified on chromosome 3, which was expected because in previous studies, chromosome 3 is a hot spot for regulation of sweet cherry fruit colour [17–19]. For example, several overlapping QTLs are on chromosome 3 [19]. The most significant SNP marker, chr3_23939472 (shared for fruit and flesh colour), collocated with many of those QTLs (Table S3) as well as with the QTL region delimited by markers CPDCT037 and EMPA014 [17]. Among known molecular markers associated with fruit colour, Pav-Rf-SSR [33] localizes at chr3_23875906–23 876 253 of the Tieton v2.0 genome, ~60 kbp from chr3_23939472. Furthermore, the single-nucleotide deletion (delA) was identified in the candidate gene *MYB10.1* responsible for blush colour [20] at the position chr3_23995550, which is ~55 kbp from chr3_23939472. Hence, chr3_23939472 is between the two mentioned markers (Pav-Rf-SSR and delA), which are currently used to predict fruit colour in sweet cherry [34]. Additional markers on chromosome 3 identified in the current analysis (chr3_19315249, chr3_24544649, chr3_25100670) also colocalized with several overlapping QTLs [17, 19] in this particularly important region (Table S3).

No haplotype block was identified for chr3_23939472; however, the closest downstream gene was transcription factor Myb10 V1–2 (FUN_015933), which belongs among the MYBs with R2R3 domains. The MYB proteins constitute a large family of transcription factors that control secondary metabolism, development, signal transduction, and resistance to biotic and abiotic stresses, among other processes [35, 36]. Several MYB proteins in many different species are also major modifiers of anthocyanin biosynthesis [18]. For example, R2R3-MYB genes responsible for anthocyanin accumulation (or its inhibition) in apples influence biosynthesis of those flavonoids in leaves and fruit skin and flesh [37]. Similarly, R2R3-MYBs activate or repress anthocyanin biosynthesis in peach [38]. Thus, the domain structure of Myb10 V1–2 indicated likely function in regulating anthocyanin biosynthesis.

The Myb10 V1–2 gene identified in this study, however, differs from the *MYB10.1* that determines fruit colour described by Jin et al. [20]. Sequencing of four yellow-fruit accessions helped to reveal the extent of deletion in the region (Fig. 4D). Notably, the assumed ~90-kbp deletion contained five highly homologous Myb genes, all possessing R2R3 domains and belonging to Clade IV of the *Prunus avium* Myb family, according to Sabir et al. [39]. Moreover, no other members of Clade IV were found in other parts of the sweet cherry genome, further indicating the importance of the region. However, the question remains: Which of the five Myb genes is/are responsible for red fruit colour? Unfortunately, the reference sequence of Tieton v2.0 suffers from mis-assembly and the reference sequence of Satonishiki contains a gap in the particular region (Fig. 4; see results for more details). To answer the question, further experiments are needed. Especially, correct assembly of the respective region would contribute valuable information.

Notably, yellow-fruited accessions possessed the deletion in homozygous constitution; whereas the parent of one of the accessions (namely SB077) was heterozygous in the region (Fig. S5). However, *fruit colour* of accession SB077 had the highest value of 9 (dark red) each year, which indicated the complete dominance of the non-deleted allele and confirmed previous findings [20].

Other candidate genes for colour traits were found close to chr1_41003304 (associated with *fruit colour*) and in haplotype blocks co-inherited with markers chr3_19315249 (associated with *fruit colour*) and chr3_24544649 (associated with *flesh colour*). Downstream of SNP chr1_41003304, anthranilate synthase beta subunit 1 (FUN_004911) was identified. The gene protein product is a part of a complex that catalyses biosynthesis of anthranilate, an intermediate in the biosynthesis of L-tryptophan, which is an amino acid scarce in cells and only infrequently used in protein synthesis [40]. However, in plants, L-tryptophan is important in regulating auxin production via the tryptophan-dependent biosynthetic pathway [41]. When exposed to naphthaleneacetic acid (NAA), sweet cherry fruits significantly upregulate transcription of key anthocyanin regulatory, biosynthetic, and transport genes, leading to an increase in anthocyanin concentration [42]. Anthocyanin accumulation after auxin application is also observed in peach [43]. The gene encoding a GATA zinc finger domain-containing protein (FUN_015375; located upstream of chr3_19315249) belongs to a family of transcription factors directing many processes in plants [44]. Notably, one of the targets of GATAs can be also MYBs, as reported in mango (*Mangifera indica* L.) [45]. In that work, the GATA-motif was identified in the light responsive element of an MiMYB1 promotor (MYB10 homolog), expression of which was correlated with anthocyanin concentration. In addition, pentatricopeptide repeat-containing protein (FUN_016023; located upstream of chr3_24544649 associated with *flesh colour*) is a member of a large family of modular RNA-binding proteins, which facilitate processing, splicing, editing, stability, and translation of RNAs [46]. Pentatricopeptide repeat proteins constitute one of the largest protein families in land plants, with more than 400 members in most species. A candidate gene in determining fruit flesh colour in melon (*Cucumis melo* L.) is CmPPR1, which likely affects carotenoid accumulation [47]. In watermelon (*Citrullus lanatus* (Thunb.) Matsum. & Nakai), ClaPPR140 cosegregates perfectly with red flesh colour [48]. In woodland strawberry (*Fragaria vesca* L.) accessions with white fruits, PPRs are among the genes significantly downregulated, compared with accessions with red fruits [49].

### Fruit firmness

A GWAS identified SNPs for fruit firmness (represented by four distinct phenotypes in this study) that were also consistent with published QTLs. The most significant SNPs for *firmness of skin* and *firmness of flesh* (chr4_16000421) and for *fruit bruiseness* (chr4_16229770) were localized between markers ss490552880 and ss490552942 that delimit the QTL for fruit firmness identified by Cai et al. [22] and collocated with QTLs qP-FF4.1^m^ identified by Calle and Wunsch [13]. Another marker associated with *firmness of skin* (chr1_31855038) was closely mapped to ss490546759 (the only marker characterizing a QTL on chromosome 1 [22]). The same SNP was also colocalized with qP-FF1.2^m^ and qP-FF1.1^m^ identified by Calle et al. [23].

The NAC transcription factor 56 (FUN_033984) emerged downstream of the most significant marker identified for both *firmness of skin* and *firmness of flesh* (chr4_16000421). The gene is among 25 candidate genes for fruit firmness located in the qP-FF4.1 locus on sweet cherry chromosome 4 [22]. In apples, NAC18.1 from the same Clade III-2 (as defined by Jensen et al. [50]) is a strong predictor of firmness at harvest and after three months of cold storage and may also function in the fruit-ripening pathway [51]. Most other firmness-linked candidate genes identified in the current study encoded cell wall-modifying proteins or influenced those proteins. For example, the BURP domain-containing protein (FUN_010126) located upstream to SNP chr2_25034310 contained a plant-specific ~230-amino acid-long protein domain. In cotton, BURP domain proteins interact with α-expansin, which can loosen cell walls [52]. In strawberries, the BURP domain-containing protein polygalacturonase 1β-like protein 3 is upregulated during ripening in soft variety Kingsberry compared with relatively firm variety Sunnyberry [53]. Additional BURP domain-containing proteins resided in a haplotype block with another marker associated with *firmness of skin* (chr1_31855038).

Polygalacturonase (PG; FUN_012870) was the only gene in the haplotype block of chr2_43495800 associated with *value of penetration*, and the SNP was localized in one of its introns. Polygalacturonase is also known as pectin depolymerase or hydrolase degrading polygalacturonans, which are significant carbohydrates in the pectin network in cell walls. Polygalacturonases soften and sweeten fruit during the ripening process, including in strawberry [54], peach [55], banana [56], and apple [57]. Moreover, the molecular marker for PG1 was validated for MAS for fruit firmness in apple [58]. Notably, cell wall-modifying enzyme activities during ripening and storage that affect fruit softening can be suppressed by exogenous application of putrescine, as shown in mango, peach, and plums [59–61]. Putrescine, one of the main plant polyamines, is formed by N-carbamoylputrescine amidase hydrolysis of N-carbamoylputrescine. The homologue of the gene (FUN_019845) was found downstream of SNP chr6_13575852 and was associated also with *value of penetration*. Three genes encoding ubiquitin-conjugating enzyme E2 (UBC) should also be considered. One was upstream of SNP chr3_19735173 (FUN_040086; associated with *value of penetration*), and two were in the haplotype block of chr7_25384148 (FUN_038919 and FUN_038920; associated with *firmness of skin* and *fruit bruiseness*). Two UBC enzymes are connected with ripening in strawberry fruit. One has a positive effect on fruit development, ripening, and softening by upregulating anthocyanin accumulation and significantly increasing expression of maturity-related genes, whereas the other increases fruit firmness [62].

While all four characteristics contributed to overall fruit firmness, they were correlated at different level. *Firmness of skin* and *firmness of flesh* shared the most significant marker and had also the highest Pearson correlation coefficient. Conversely, *value of penetration*, the only firmness trait measured under standard laboratory conditions, showed lower correlation to all other traits (Fig. S6). The distinctness of *value of penetration* might be connected to difference between precise quantitative values and qualitative evaluation of other traits at 9-pointed sensory scale despite it was done by skilled experts. *Fruit bruiseness* was linked to *firmness of skin* by sharing one of the significant SNPs. However, it had a similar correlation with *firmness of flesh* and both characteristics (*firmness of skin* and *firmness of flesh*) are expected to contribute to *fruit bruiseness*. The traits classified as fruit firmness are influenced by such parameters as water content [63], chemical composition of cell wall [64] or cell number and size (as firmness negatively correlates with fruit weight [21]). These components might contribute to individual firmness traits by varying degrees resulting in the distinct loci identified in GWAS.

### Fruit size and weight

Traits *fruit length*, *fruit width*, *fruit thickness* and *weight of fruit* were used to evaluate fruit size and weight. Although all four phenotypes were highly correlated, SNPs usually differed among the parameters. Such differences were not surprising because size characteristics were not in “mutually fixed” ratios. Instead, different ratios reflected heterogeneous fruit shapes. Nevertheless, three markers from chromosome 2 associated with *fruit length*, *fruit thickness* and *weight of fruit* collocated in a single haplotype block, indicating it was a very strong candidate to determine fruit size. Overall, SNPs for individual size-related traits were identified on all chromosomes except chromosome 7, although the most significant associations were found on chromosomes 1 and 2. This result is consistent with known QTLs associated with fruit size and weight. The SNP marker with the lowest *p*-value (chr2_31850964; associated with *fruit width*) was collocated with multiple QTLs, i.e. qP-FW2.1^m^ [13] and FW_G2a [25], and QTLs defined by markers Ma069a–MA005c and CPSCT038–BPPCT034 [24]. In addition, the haplotype block that included SNPs markers chr2_29776624 (associated with *fruit length*), chr2_29776725 (the most significant for *fruit thickness*), and chr2_29787028 (the most significant for *weight of fruit*) was in regions of qP-FW2.1^m^ [13] and the QTL defined by Ma069a and MA005c markers [24]. Similarly, locations of SNPs identified on chromosome 1 were consistent with positions of published QTLs [13, 23, 25].

The cell number regulator gene *PavCNR12* has been proposed [26] as a candidate gene for a fruit weight QTL [24]. However, the gene (FUN_010963; localized at chr_2:31917104–31 920 001 in Tieton reference v2.0) was separated by ~66 kb from the closest SNP significant in the GWAS, namely chr2_31850964. Whereas the largest haplotype block (307 SNPs, 58.6 kb) was co-inherited with the respective SNP in the current study, it included only three genes, and *PavCNR12* was not among them. Nevertheless, another promising candidate appeared among the three collocated genes. Nucleoside diphosphate kinase 1 (NDPK1; FUN_010956) has a major role in the biosynthesis of nucleoside triphosphates (other than ATP) [65]. Nucleoside diphosphate kinase 1 is associated with expansions in different tissues, and its expression increases during the growth of grape berry [66]. Its homologue NDPK2 contributes to fruit development and maturity in *Arabidopsis thaliana* (L.) Heynh. [67]. To determine which gene contributes (or whether both do) to size differences associated with the particular locus, additional analyses are needed. Nevertheless, this study confirmed a strong association of the respective locus with fruit size and also provided additional marker(s) tightly linked with the trait.

A nearby haplotype block (290 SNPs) with SNP markers chr2_29776624, chr2_29776725, and chr2_29787028 (all strongly associated with fruit size characteristics) provided additional candidate genes. Cyclin-U4–1-like (FUN_010712) is involved in the G1/S phase transition during cell division, and it has significantly higher expression in a bottle gourd (*Lagenaria siceraria* (Molina) Standl.) accession with big fruits than in one with small fruits [68]. Similarly, Cyclin-U4–1 is among genes downregulated in transgenic tomato (with reduced level of cytokinins), which has smaller fruits than those of the wild type [69]. Another gene in the haplotype block encoded endoglucanase 4 (FUN_010713). Endoglucanases have roles in fruit growth and ripening, for example, in peach [70]. Additional SNPs were also localized near other genes linked to cell wall (re)organization that enables cell expansion; for example, putative wall-associated receptor kinase (FUN_032569) and beta-glucosidase 11 (FUN_020197).

Another gene potentially associated with fruit size and weight was E3 ubiquitin-protein ligase RGLG5 (FUN_003761), which was found in a haplotype block (221 SNPs) surrounding another significant SNP marker, chr1_33914270 (associated with *fruit width* and *weight of fruit*). The RGLG5, together with RGLG1, mediates the ubiquitination and subsequent degradation of phosphatase PP2CA, a major inhibitor of ABA signalling [71]. Liao [72] showed the importance of synchronized action of plant hormones in nonclimacteric fruit growth and ripening. At the beginning of *Fragaria vesca* fruit development, auxins and gibberellins are responsible for fruit growth (supporting both cell division and enlargement), and ABA accumulation is suppressed. With onset of fruit ripening, auxin and gibberellin levels decrease, leading to an abrupt increase in ABA that drives fruit ripening. Therefore, ABA may be negatively correlated with fruit size [72]. Because RGLG5 can influence the level of this crucial plant hormone, the encoding gene is also a promising candidate for fruit size regulation.

## Conclusion

A set of highly significant SNPs associated with harvest time and fruit colour, firmness, and size, as well as haplotype blocks, were identified in a population of sweet cherry accessions. Allele effects of individual SNPs showed clearly their potential for MAS, because allelic combinations of particular SNPs were frequently linked with different phenotypes. Associations between phenotypic characteristics and particular allelic combinations were confirmed in an independent breeding population. The most significant SNPs were successfully linked to previously known QTLs for respective traits. For many SNPs, meaningful candidate genes were identified that were co-inherited with them in haplotype blocks, which supported further use of SNPs in MAS, and provide a framework to characterize genes controlling particular traits and thus improve sweet cherry. Moreover, the deep sequencing of 299 accessions provides valuable resources for further analysis of the sweet cherry genome.

## Materials and methods

### Plant materials

In GWASs, 235 sweet cherry accessions (Supplementary Data) were used from the Collection of Genetic Resources (CGR) of the Research and Breeding Institute of Pomology Holovousy Ltd. (RBIPH). Accessions included commercial and older historic cultivars, important breeding hybrids, and traditional Czech landraces. Germplasms were established at RBIPH in 1989. Holovousy (50.3681°N, 15.5731°E) has an annual average temperature of 8.4°C and annual precipitation of 663.5 mm [73]. The collection (three plants representing each cultivar) on vigorous mazzard seedling rootstock was planted with 6.0 × 6.0-m spacing at an altitude of 320 m. Breeding materials (BM), a set of 64 new sweet cherry accessions, on semidwarf rootstock Gisela 5 were planted with 5.0 × 1.5-m spacing in 2006 to 2009. The younger set of breeding trees was not used in GWASs but to verify GWAS results. All trees were trained to a central leader with evenly distributed and well-developed scaffold branches. Annual pruning was performed in March and April to develop and maintain tree size and shape. Strips under tree crowns were maintained weed-free using herbicides, and plantations were not irrigated. Standard pest management and fertilization practices were followed.

### Phenotype characterization

All accessions were phenologically and phenotypically evaluated by using modified descriptors for sweet cherries [74, 75] from 2012 to 2020. Assessments of CGR and BM tree sets were performed independently in parallel by two groups of skilled evaluators using the same 11 descriptors of *time of harvest*, *fruit colour, flesh colour, firmness of skin, firmness of flesh, value of penetration, fruit bruiseness, fruit length, fruit width, fruit thickness,* and *weight of fruit.* Assessment of fruits was done directly after sampling and no cooling was applied on fruit before evaluation. Fruit was kept in ambient temperature of 24°C.

***Time of harvest** -* date, when mature fruits were collected. Determined with respect to the first ripening reference cultivar Kisinevskaja (SA993) to minimize seasonal perturbations, mainly weather, over the years. All values for SA993 were set to “0” and values for other accessions were standardized to it. Kisinevskaja usually ripens 0–2 days before well-known cultivar Früheste der Mark (syn. Krüppers Frühkirsche or Rychlice nemecka).

***Fruit colour*** and ***flesh colour*** were visually determined on ten fruits by skilled evaluators using 9-point classification scale (Fig. S11, Table S6).

***Firmness of skin, firmness of flesh,*** and ***fruit bruiseness*** were determined by skilled evaluators using 9-point classification scale according to Paprštein et al. [74] (Table S7). *Firmness of skin* was evaluated as sensory assessment of the firmness of the fruit skin when biting into the whole fruit (1-very soft; 9-very firm). *Firmness of flesh* represented sensory assessment of the firmness of the pulp when biting into the flesh (1-very soft; 9-very firm). *Fruit bruiseness* was assessed as the fruit resistance to bruising due to compression by squeezing the fruits between the fingers. Squeezing with fingers do not cause visible damage or change in colour of the fruit. *Fruit bruiseness* was sensory evaluated as a measure of fruit elasticity or how solid the fruit appears to the touch (1-very high sensitivity to bruiseness; 9- absent of bruiseness). Each characteristic was evaluated for fifteen fruits.

***Value of penetration*** for CGR set was measured by compression on cheek (years 2015–2020 only) and on the right side of the fruit suture with a 3 mm tip of FTA-Fruit Texture Analyzer, (Guss, South Africa), and expressed in kg.cm^−2^. An average value of ten fruits per cultivar was recorded. Fruits from BM set were measured in years 2012–2020 manually using a digital firmness tester AGROSTA 100X (Agro technologie, France) with Tip of 25 (6 mm) and expressed in index value of firmness (1–100). Five fruits per genotype were measured by compression on cheek, on the left and the right side of the fruit suture and average value of penetration was noted. Undamaged fruit was used for each penetration experiment.

***Weight of fruit*** including the stalk was determined with a digital balance (RADWAG WLC 6/A2/C/2, d = 0.1 g, Poland) and calculated as an average from a weight of one hundred cherry fruits. ***Fruit length***, ***fruit width**,* and ***fruit thickness*** were measured in millimetres with a calliper and calculated as an average from 10 fruits (Fig. S12).

Final values of each phenotype and sample were means of values over all trees and years (Supplementary Data). Those values were used in subsequent analyses. The Ggpairs() function of the GGally package in R v4.0.0 (https://www.r-project.org/) was used to determine and display Pearson correlation coefficients between phenotypes.

### DNA extraction, sequencing, and simple sequence repeat analysis

Genomic DNA was isolated from 100 mg of shoot phloem using an Exgene Plant SV isolation kit (GeneAll) according to the manufacturer’s instructions. Bark from 1- to 2-year-old shoots was peeled off by scalpel, and phloem was scraped and then homogenized in liquid nitrogen using a mortar and pestle. An Invitrogen Qubit Fluorometer was used to assess DNA quality. The 50 ng DNA was fragmented in 50 μl using a Bioruptor Plus (Diagenode); five cycles were run for 30 s at the high setting. Libraries for sequencing were prepared using a NEBNext Ultra™ II DNA Library Prep Kit for Illumina (New England Biolabs) with three cycles of amplification. Libraries were sequenced on a NovaSeq 6000 (Illumina), producing 2 × 150-bp paired-end reads. Simple sequence repeat markers were used to analyse pedigrees of yellow-fruited cherries following the protocol of Clarke and Tobutt [76].

### SNP calling and data processing

For SNP calling, the reference sequence from the *P. avium* “Tieton” Genome v2.0 [2] was used. Raw data were trimmed for low-quality bases and adapter sequences using Trimmomatic v0.32 [77]. Trimmed reads for each accession were mapped to the reference sequence with BWA-MEM v0.7.15 [78], and duplicated reads were removed using the Picard tool *MarkDuplicates* (http://broadinstitute.github.io/picard/). Resulting alignments were processed using GATK v4.0.12.0 [79] software with HaplotypeCaller and CombineGVCFs tools to call SNPs. Resulting SNPs were filtered according to the best practice of hard filtering (QD < 2.0, QUAL <30.0, SOR > 3.0, FS > 60.0, MQ < 40.0, MQRankSum < −12.5, ReadPosRankSum < −8.0, and DP < 8.0). Samples missing more than 10% SNPs and variants missing more than 5% SNPs were excluded from further analysis using PLINK v1.90 [80]. Missing calls were imputed using the software Beagle v5.2 [81], and all SNPs with minor allele frequency less than 1% and Hardy–Weinberg Equilibrium at *p* < 1e^−6^ were removed.

### Genetic structure analysis and genome-wide association studies

Principal component analysis was used to determine number of subpopulations with GAPIT3 software (Genome Association and Prediction Integrated Tool) [82]. The PCA plots were generated using Rstudio (https://www.rstudio.com/). Kinship matrix was generated from the SNP data set by the VanRanden algorithm method [83] and visualized as a heat map.

Associations between molecular markers and phenotypic data were computed for the CGR set in GAPIT3. Two models were used in a preliminary analysis: Bayesian-information and linkage-disequilibrium iteratively nested keyway (Blink) and Fixed and random model circulating probability unification (FarmCPU). Model performance was compared based on a genomic inflation factor (lambda) calculated from *p*-values obtained from association analysis. The FarmCPU model was most suitable for the data set. According to BIC values generated using GAPIT, the PCA parameter in following analysis was set to 0. Final GWASs for all phenotypes were performed using FarmCPU, and markers above the 5% Bonferroni multiple test threshold values were considered significant. Effects of alleles for each SNP marker were determined based on phenotype values, and SNP calls and corresponding box plots were created using Rstudio.

### Identification of haplotype blocks and characterization of candidate genes

The software PLINK v1.90 was used to select 100-kbp-long regions surrounding associated SNP markers, including allele combinations for all filtered SNP markers. Haploview v4.2 [84] was used to compute LD statistics and define haplotype blocks around each SNP marker. All genes in haplotype blocks on the Tieton reference sequence were considered as candidates and subsequently annotated. If no gene was present in a particular haplotype block, the closest down- and up-stream genes were considered as candidates. Gene function was identified based on 1) mapping to KEGG orthologs (provided at https://www.rosaceae.org/Analysis/9262820); 2) a BLASTP search against nonredundant databases of Viridiplantae proteins at https://blast.ncbi.nlm.nih.gov; and 3) assignment to protein families using InterProScan at https://www.ebi.ac.uk/interpro/.

### Comparison to published quantitative trait loci

Locations of significant SNPs identified in GWASs were compared with positions of known QTLs. To find positions of SSR, CAPS, and SNP markers used to define QTLs, relevant sequence information on particular markers and genetic maps was obtained from www.rosaceae.org, www.ncbi.nlm.nih.gov, Cherry Genome DataBase (kazusa.or.jp), or other publications (i.e. original citations, especially in cases of SSR markers). Sequences of primers or amplicons (in cases of SSR and CAPS markers) and surrounding sequences (in cases of SNPs; min. 500 bp) were searched by BLASTN against the *P. avium* “Tieton” v2.0 genome [2] at www.rosaceae.org to obtain unique positions of analysed markers. In the absence of more specific markers, the nearest outer markers delineating a QTL in genetic maps were used to assign the region of a particular QTL.

### Characterization of a deletion in the MYB region

To characterize the deletion around the *MYB10.1* gene determining yellow colour of sweet cherry fruits, a region of chromosome 3 was studied in detail. Paired-end reads from accessions with yellow fruits (SB083, SB124, SB134, SB377), four randomly selected accessions with red fruits (SA118, SA245, SA999, SB015), and Tieton [2] were mapped using BWA-MEM to the Tieton and Satonishiki reference sequences. Duplicated reads were removed using the Picard tool *MarkDuplicates*. Non-uniquely mapped reads were filtered out using SAMtools v1.9 [85] with the following \aa \aa \aa command: “samtools view -@ 6 -q 1 -F 4 -F 256 -h <Input.bam>|grep -v -E -e ‘\bXA:Z:’ -e ‘\bSA:Z:’|samtools view -b -T <Reference.fasta> - > <Output_uniq.bam>”. For each position on a reference sequence, read depth was calculated using the BEDTools v2.26.0 [86] subcommand genomecov. Read depth was recalculated for 1000-bp or 500-bp windows and visualized as line graphs using ggplot packages in R software.

## Acknowledgments

We thank Petra Chudobová, Lenka Křivohlávková, Lenka Tůmová, Lucie Zálešáková, and Helena Tvardíková for outstanding technical assistance. This work was supported by the Ministry of Agriculture of the Czech Republic (project QK1910290). Computational resources were supplied by the project “e-Infrastruktura CZ” (e-INFRA CZ LM2018140) supported by the Ministry of Education, Youth and Sports of the Czech Republic.

## Author Contributions

J.B., J.C., L.Z., P.S., R.C. designed the research; K.H., J.C., P.S., J.S., R.C. performed the experiments; K.H., J.B., J.C., R.C. analysed and interpreted the data; K.H., J.C., J.B., P.S., J.S. wrote the manuscript; all authors revised and approved the manuscript.

## Data availability

Raw data have been submitted to the Sequence Read Archive (SRA) under study number PRJNA813711.

## Conflict of interests

The authors declare no conflicts of interest.

## Supplementary data

Supplementary data is available at *Horticulture Research* online.

## Supplementary Material

Web_Material_uhac233
