# Supplementary material for: High-resolution genome-wide association study of a large Czech collection of sweet cherry (Prunus avium L.) on fruit maturity and quality traits
Source: Hortic Res. 2022 Oct 19;10(1):uhac233. doi: 10.1093/hr/uhac233 (PMC9832837; doi:10.1093/hr/uhac233)
Supplement: Web_Material_uhac233 [file web_material_uhac233.zip › Supplementary Figures and Tables.pdf]

Supplementary Figures

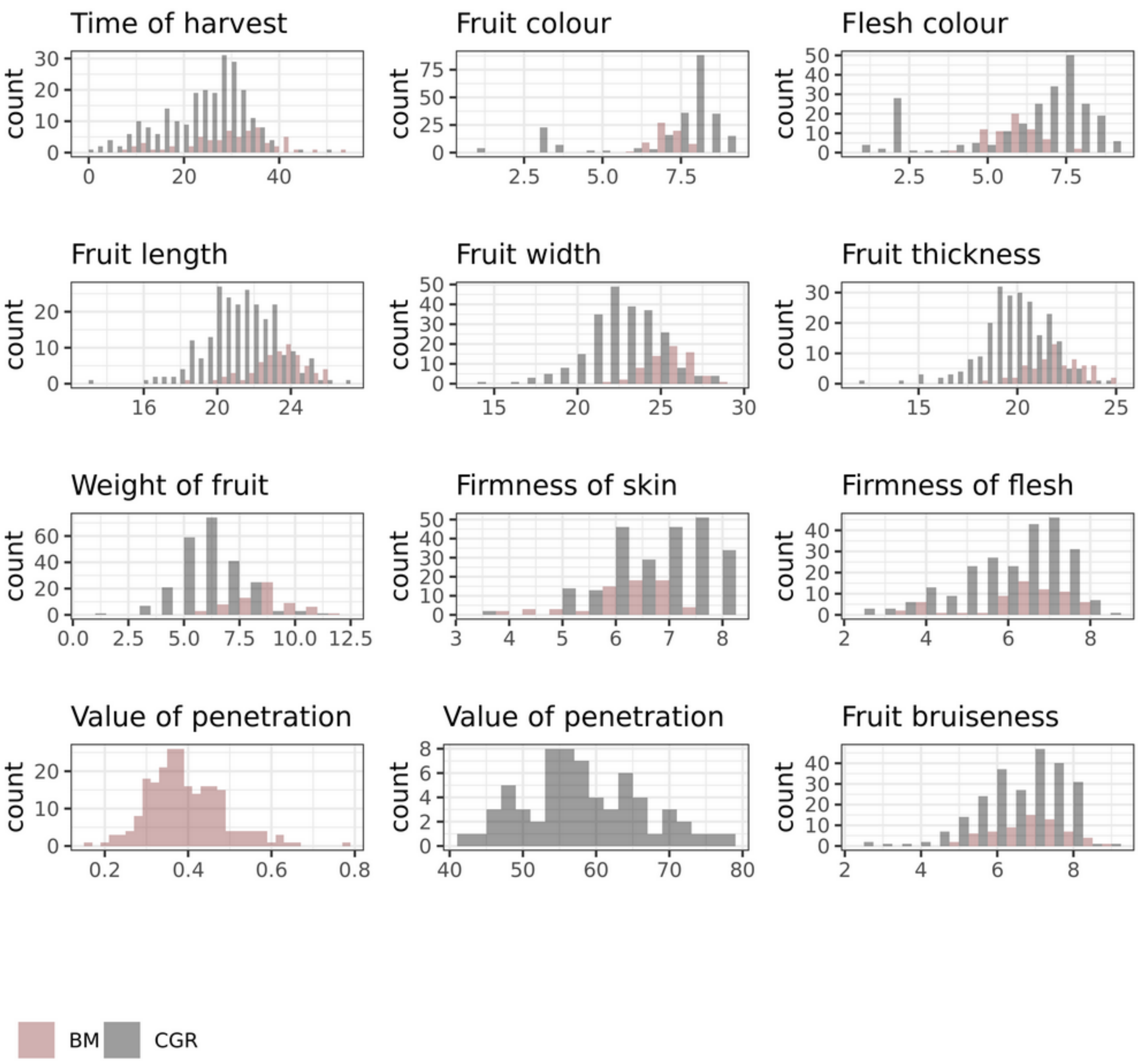

Fig. S1: Distribution of phenotypic values (x-axis) between CGR and BM population. Values on x-axis are in case of *time of harvest* in days after ripening of reference cultivar Kisinevskaja (SA993); in case of *fruit colour, flesh colour, firmness of skin, firmness of flesh*, and *fruit bruiseness* correspond to 9-point classification scale used for their evaluation; *fruit length, fruit width*, and *fruit thickness* are specified in millimetres; *weight of fruit* is in grams; and *value of penetration* is presented in $kg.cm^{-2}$ for CGR, and in scale 1-100 for BM population, respectively.

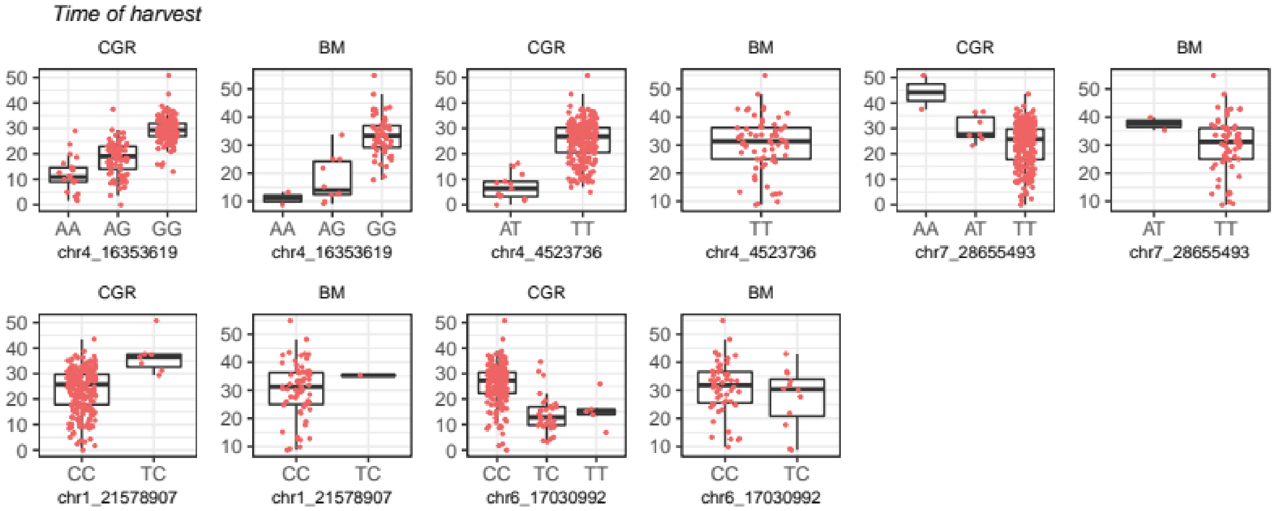

Fig. S2: Allele effect for harvest traits. All significant SNP markers above Bonfernonni's thresholds for *time of harvest* are presented. Box plots show phenotypic values for particular trait associated to individual combinations of alleles. Each plot represent association for single SNP marker either for CGR (left) or BM (right) population. The SNPs are organized according to significance from the most to less significant one for each trait. Phenotypical value is number of days after reference cultivar Kisinevskaja (SA993).

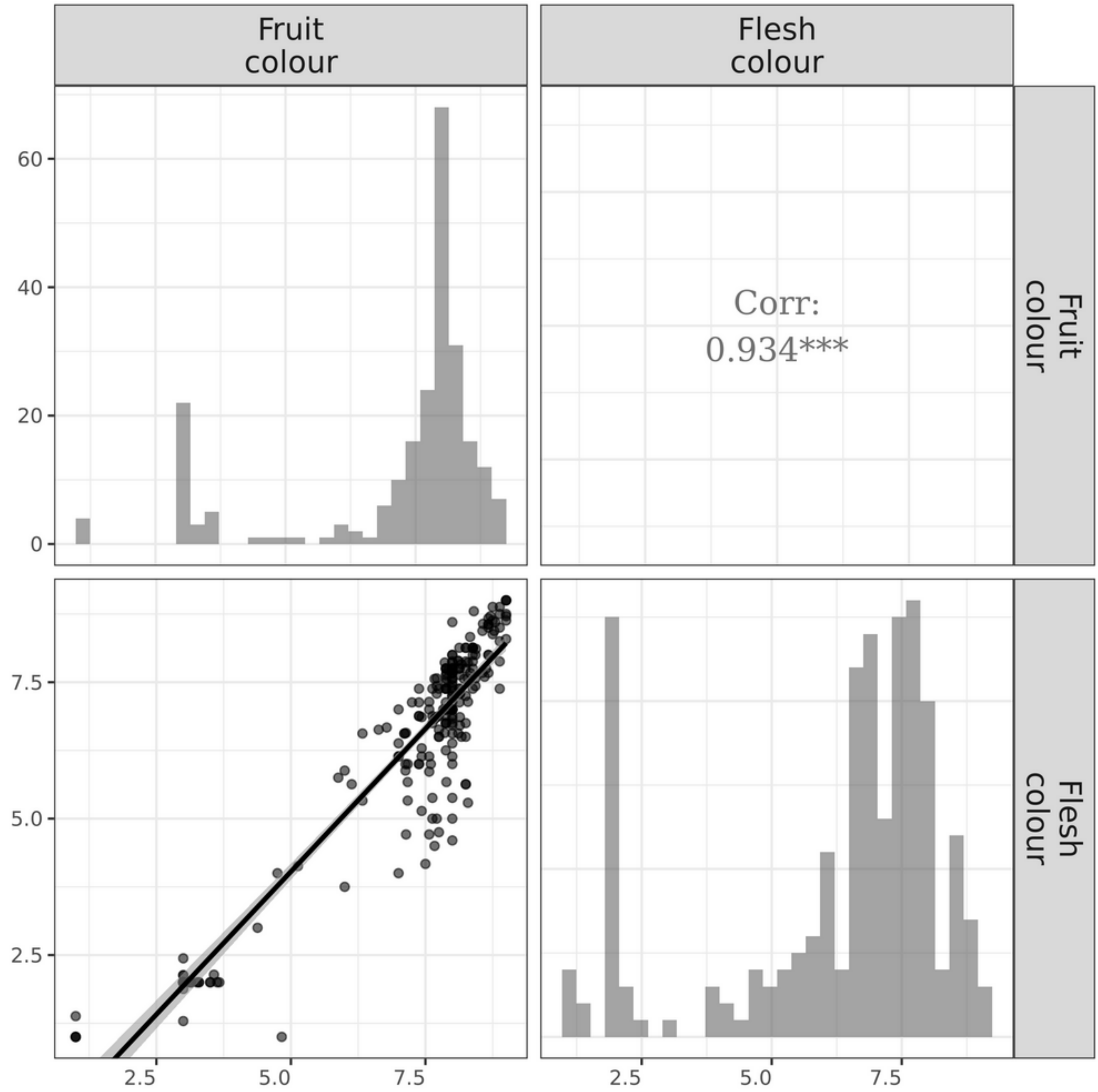

Fig. S3: Relationship of phenotypic values among colour characteristics. For each pair of traits, the figure displays Pearson correlation coefficient (upper right corner), density plot (diagonal) and scatter plot (lower left corner) calculated from the phenotypic values of individual accessions. Lower and higher values correspond to yellow and red fruits, respectively.

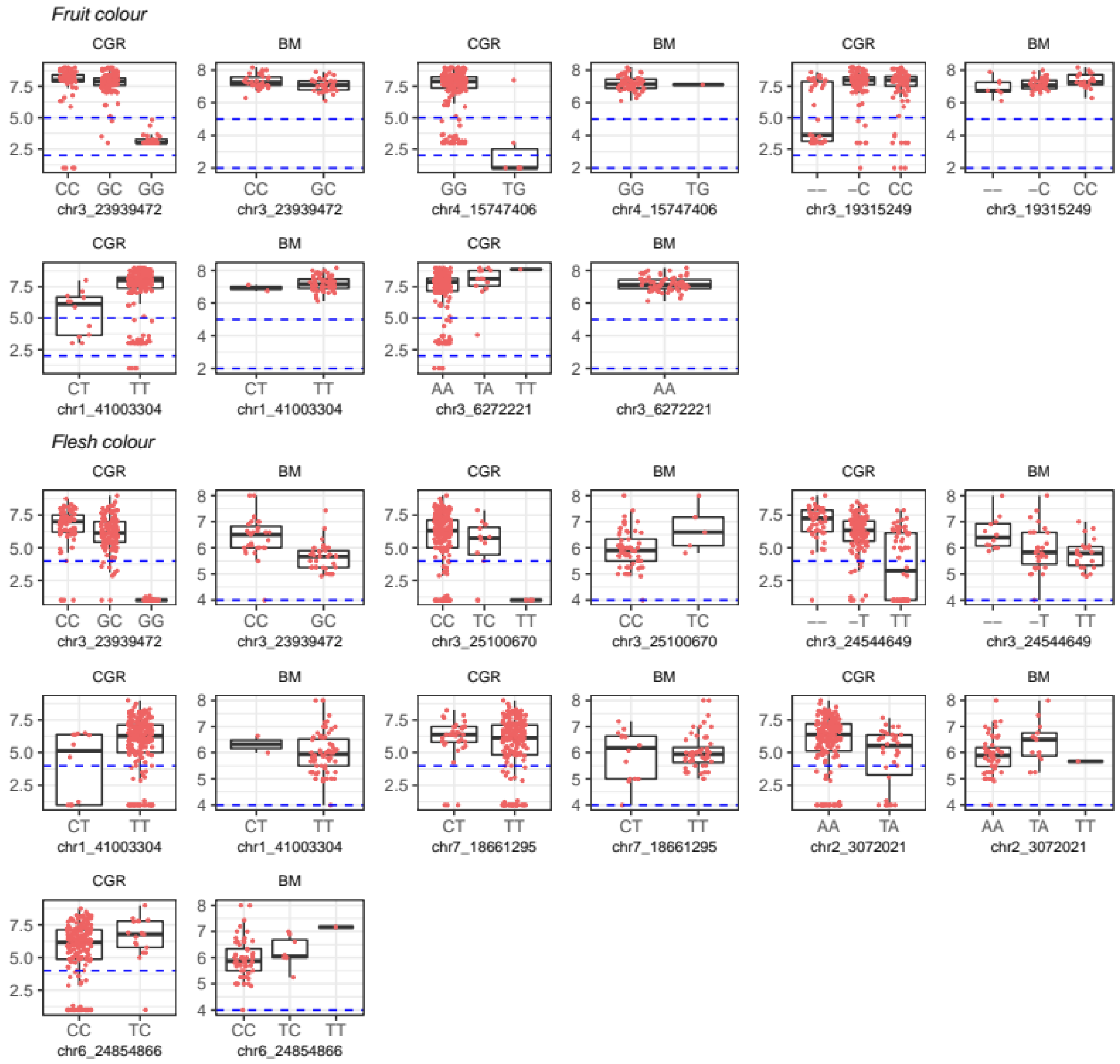

Fig. S4: Allele effect for colour traits. All significant SNP markers above Bonfernonni's thresholds for *fruit colour* and *flesh colour* are presented. Box plots show phenotypic values for particular trait associated to individual combinations of alleles. Each plot represent association for single SNP marker either for CGR (left) or BM (right) population. The SNPs are organized according to significance from the most to less significant one for each trait. Phenotypical values between the two blue dash lines represent accessions with marble *fruit colour*. Lower and higher values correspond to yellow and red fruits, respectively. Blue dash line in *flesh colour* divided accessions with yellow (under line) and red phenotype. Values on x-axes correspond to 9-point classification scale.

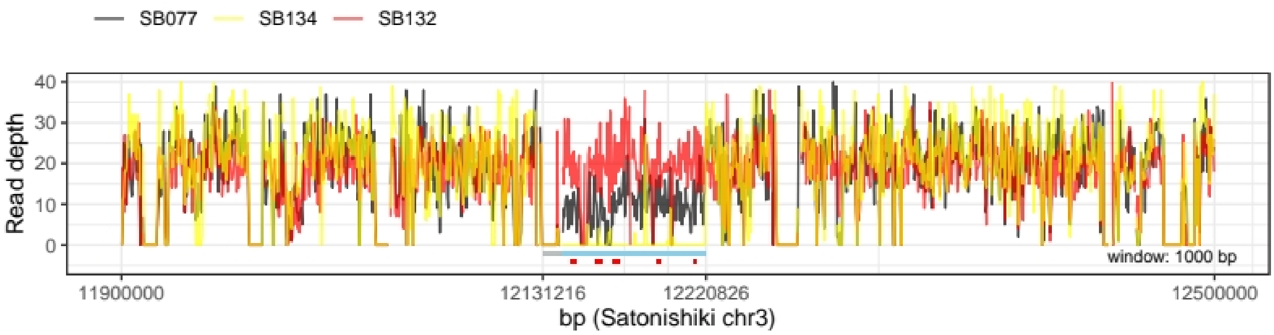

Fig. S5: Analysis of deletion in MYB10 region for yellow-fruited cherry and its parent. Read depth (window 1 kbp) of three accessions: yellow-coloured fruits (SB134), red-coloured fruits (SB132) and dark-red-coloured fruits (SB077, parent for SB134). Reads ware mapped to Satonishiki reference. Blue line highlight deletion found in accessions with yellow-coloured fruits and red lines below marked MYB genes.

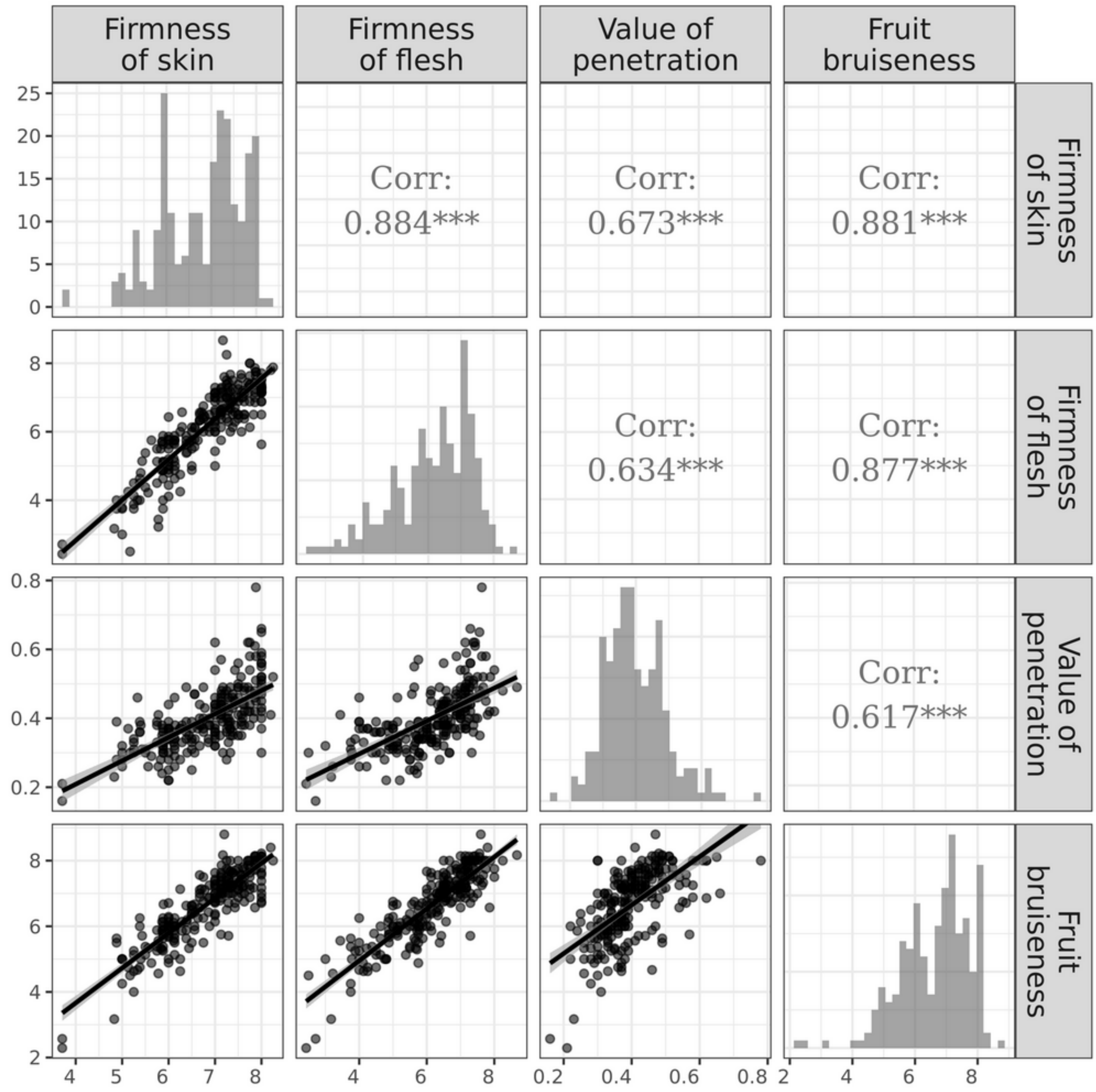

Fig. S6: Relationship of phenotypic values among firmness characteristics. For each pair of traits, the figure displays Pearson correlation coefficient (upper right corner), density plot (diagonal) and scatter plot (lower left corner) calculated from the phenotypic values of individual accessions. Values for *firmness of skin*, *firmness of flesh*, and *fruit bruiseness* correspond to 9-point classification scale used for their evaluation; *value of penetration* is presented in kg.cm^{-2}.

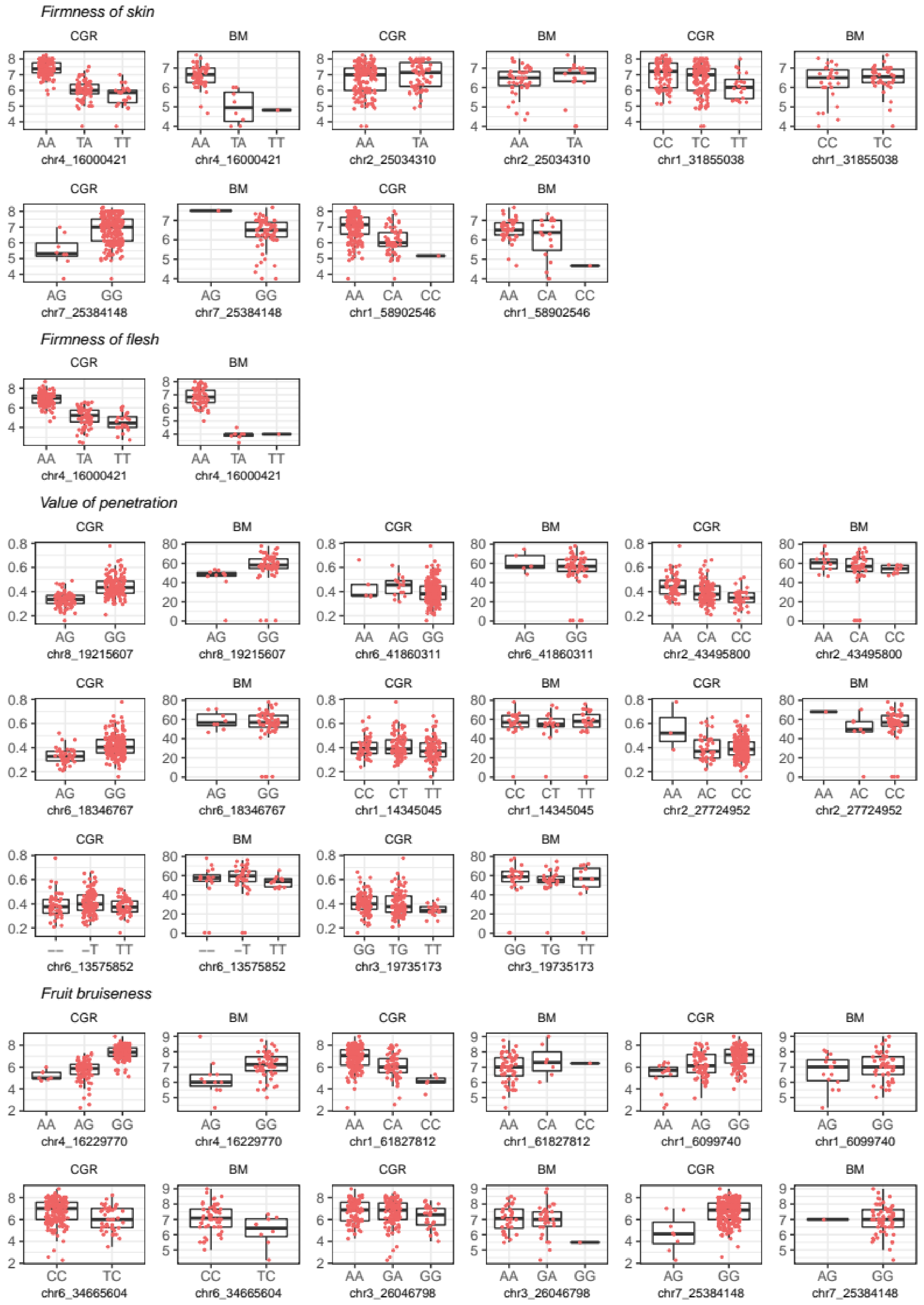

Fig. S7: Allele effect for firmness traits. All significant SNP markers above Bonfernonni's thresholds for *firmness of skin*, *firmness of flesh*, *value of penetration*, and *fruit bruiseness* are presented. Box plots show phenotypic values for particular trait associated to individual combinations of alleles. Each plot represent association for single SNP marker either for CGR (left) or BM (right) population. The SNPs are organized according to significance from the most to less significant one for each trait. Values on y-axes in *firmness of skin*, *firmness of flesh*, and *fruit bruiseness* correspond to 9-point classification scale used for their evaluation; *value of penetration* is presented in kg.cm^{-2} for CGR, and in scale 1-100 for BM population, respectively.

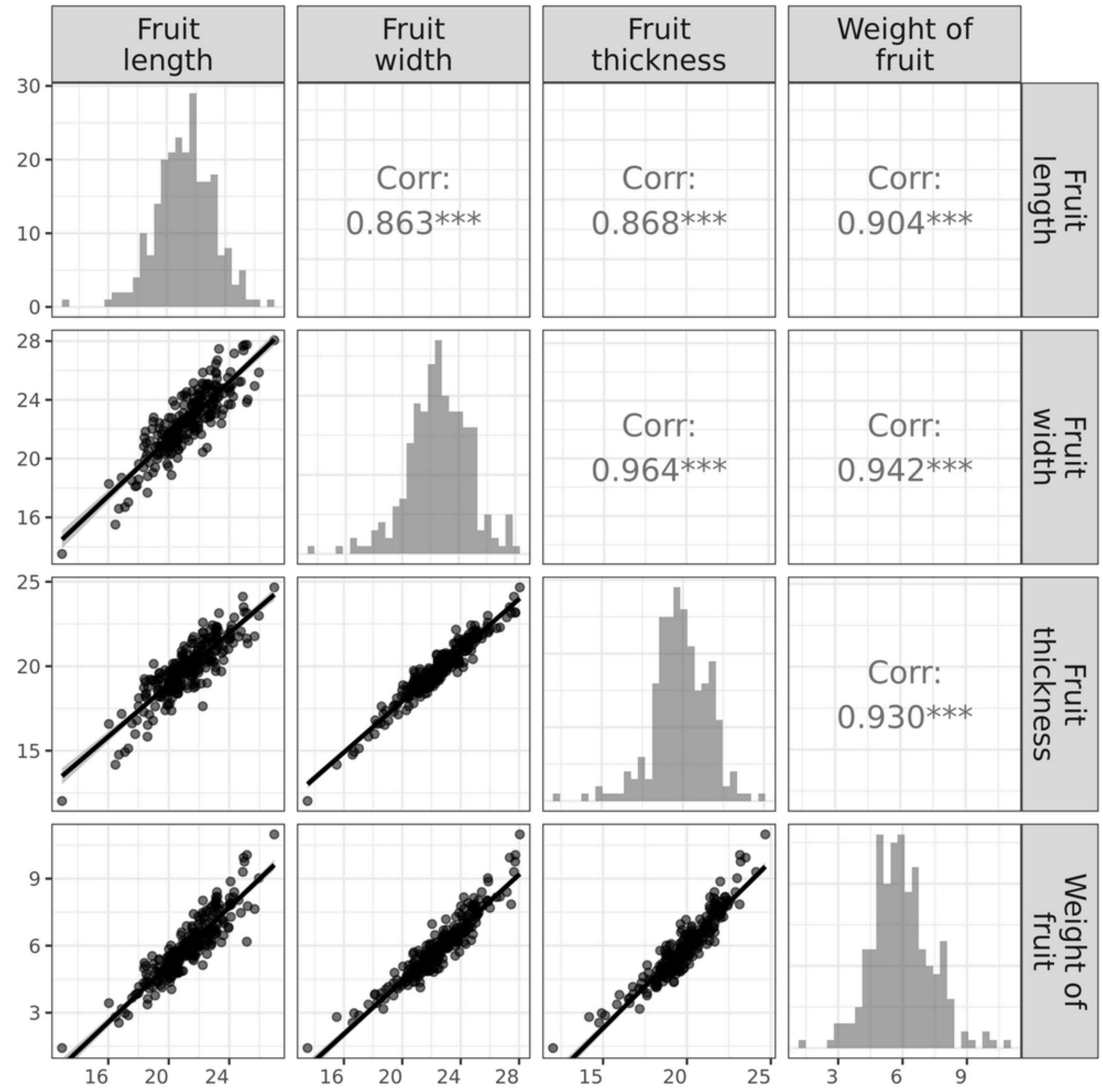

Fig. S8: Relationship of phenotypic values among size characteristics. For each pair of traits, the figure displays Pearson correlation coefficient (upper right corner), density plot (diagonal) and scatter plot (lower left corner) calculated from the phenotypic values of individual accessions. *Fruit length, fruit width*, and *fruit thickness* are specified in millimetres; *weight of fruit* is in grams.

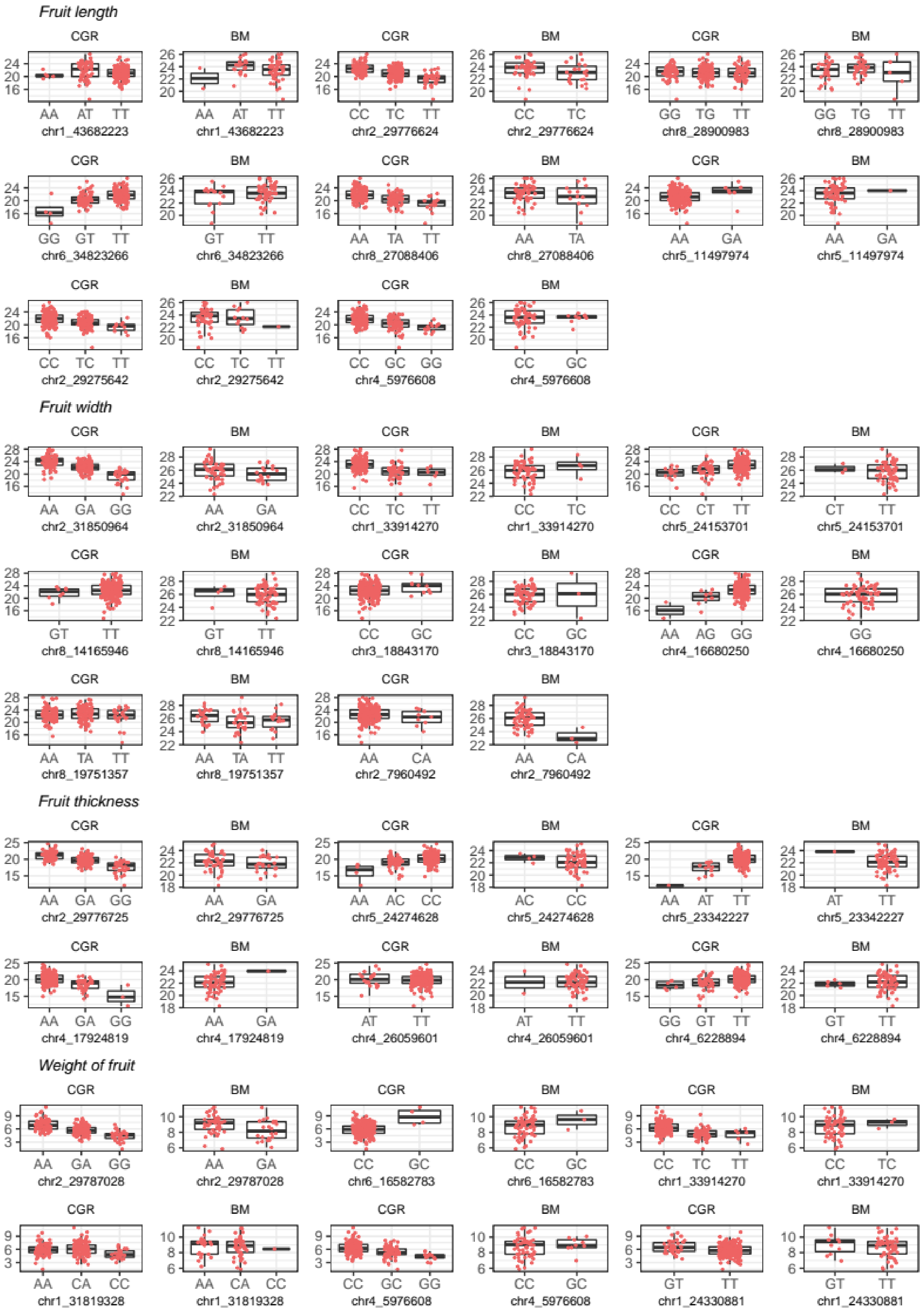

Fig. S9: Allele effect for size traits. All significant SNP markers above Bonfernonni's thresholds for *fruit length*, *fruit width*, *fruit thickness*, and *weight of fruit* are presented. Box plots show phenotypic values for particular trait associated to individual combinations of alleles. Each plot represent association for single SNP marker either for CGR (left) or BM (right) population. The SNPs are organized according to significance from the most to less significant one for each trait. *Fruit length, fruit width*, and *fruit thickness* are specified in millimetres; *weight of fruit* is in grams.

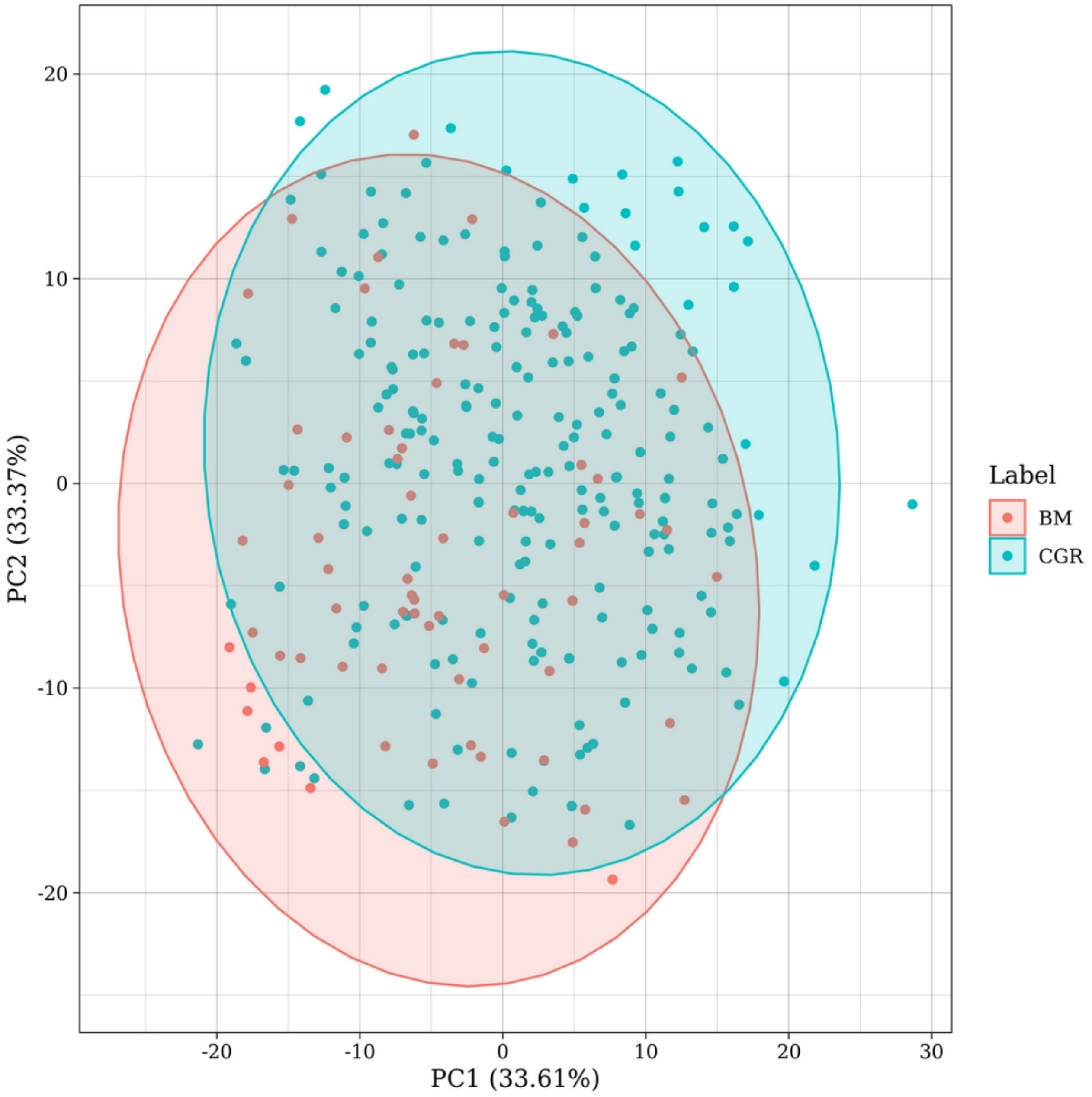

Fig. S10: PCA analysis. Plot of the first two Principal Components identified among 299 accessions (CGR and BM) based on 1,767,106 SNPs.

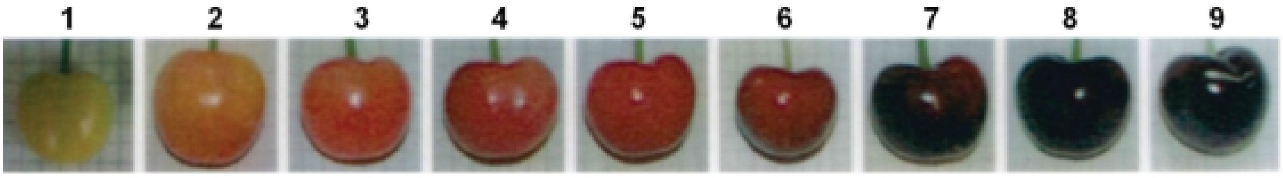

Fig. S11: A colour chart for evaluating cherry fruit skin

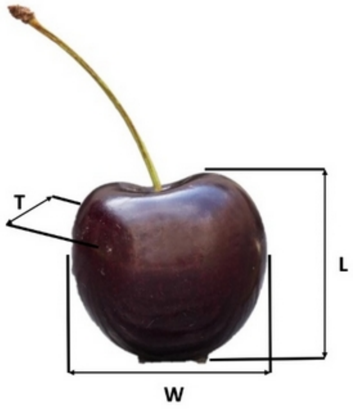

Fig. S12: Dimensional characteristics of sweet cherry fruit. L - length; W - width; T - thickness.

Supplementary Tables

Tab. S1: Comparison of lambda factor for FarmCPU and Blink models for all traits.

	Model	
**Characteristic**	**Blink**	**FarmCPU**
**Time of harvest**	1.32	1.01
**Fruit colour**	1.62	0.85
**Flesh colour**	1.16	0.91
**Firmness of skin**	1.37	1.01
**Firmness of flesh**	1.09	0.83
**Value of penetration**	1.13	0.98
**Fruit bruiseness**	1.14	0.89
**Fruit length**	1.35	1.02
**Fruit width**	1.28	1.06
**Fruit thickness**	1.17	1.05
**Weight of fruit**	1.21	1.08

Tab. S2: SNP markers associated with *time of harvest*, phenotype mean values for genotypes, its analysis of variance, and mapping on QTLs

Time of harvest												
						Phenotype mean values for alleles			Analysis of variance			
SNP	CHR	Position	P-value	MAF	Alleles	0/0	0/1	1/1	F	P-value	F critical	Mapping on QTLs
chr4_16353619	4	16,353,619	7.21E-26	0.26	A/G	11.86	18.35	29.35	147.94	4.98E-42	3.04	a
chr4_4523736	4	4,523,736	2.57E-21	0.03	A/T		7.01	24.97	68.60	9.63E-15	3.88	b, c
chr7_28655493	7	28,655,493	6.18E-10	0.02	A/T	44.13	29.93	23.61	7.75	5.55E-04	3.03	
chr1_21578907	1	21,578,907	1.03E-09	0.01	T/C		36.73	23.58	16.85	5.59E-05	3.88	d
chr6_17030992	6	17,030,992	1.48E-08	0.09	T/C	15.58	14.07	25.92	39.50	1.75E-15	3.03	

a) HD_Y4_56 (14.8-19.7 Mbp) [12]; **b)** AMPA100-PMS3 (2.1-5.0 Mbp) [10]; **c)** qP-MD4.1^{m} (1.3-10.4 Mbp) [13]; **d)** qP-MD1.1^{m} (18.8- 37.8 Mbp) [13]

Tab. S3: SNP markers associated with colour characters, phenotype mean values for genotypes, its analysis of variance, and mapping on QTLs

**Fruit colour**												
						Phenotype mean values for alleles			Analysis of variance			
SNP	CHR	Position	P-value	MAF	Alleles	0/0	0/1	1/1	F	P-value	F critical	Mapping on QTLs
chr3_23939472	3	23,939,472	1.37E-41	0.41	G/C	3.24	7.77	7.69	192.43	6.49E-50	3.03	a-g
chr4_15747406	4	15,747,406	5.31E-30	0.01	T/G		2.50	7.28	43.40	2.96E-10	3.88	
chr3_19315249	3	19,315,249	1.01E-14	0.46	-/C	5.34	7.65	7.38	26.52	4.28E-11	3.03	a, e, g
chr1_41003304	1	41,003,304	2.25E-11	0.03	C/T		5.38	7.26	11.48	8.25E-04	3.88	
chr3_6272221	3	6,272,221	4.76E-09	0.03	T/A	8.88	7.94	7.12	1.64	1.96E-01	3.03	
**Flesh colour**												
						Phenotype mean values for alleles			Analysis of variance			
SNP	CHR	Position	P-value	MAF	Alleles	0/0	0/1	1/1	F	P-value	F critical	Mapping on QTLs
chr3_23939472	3	23,939,472	3.15E-35	0.41	G/C	2.02	6.83	7.10	166.99	1.37E-45	3.03	a, h-l
chr3_25100670	3	25,100,670	3.71E-22	0.04	T/C	1.09	5.88	6.42	13.73	2.32E-06	3.03	a, h-j, l
chr3_24544649	3	24,544,649	3.58E-16	0.37	-/T	7.56	6.76	4.38	52.09	2.13E-19	3.03	a, h-l
chr1_41003304	1	41,003,304	1.60E-14	0.03	C/T		4.60	6.39	8.21	4.56E-03	3.88	
chr7_18661295	7	18,661,295	3.35E-12	0.08	C/T		6.78	6.21	2.20	1.39E-01	3.88	
chr2_3072021	2	3,072,021	2.14E-11	0.07	T/A		5.34	6.46	8.19	4.59E-03	3.88	
chr6_24854866	6	24,854,866	6.07E-11	0.04	T/C		7.30	6.22	4.05	4.54E-02	3.88	

a) CPDCT037-EMPA014 (17.5-34.5 Mbp) [17]; **b)** qP-SC3.1-2017 (21.5-24.1 Mbp) [19]; **c)** qP-SC3.2-2017 (21.6-24.9 Mbp) [19] ; **d)** qP-SC3.1-2018 (21.5-31.7 Mbp) [19]; **e)** qP-SC3.2-2018 (12.0-30.0 Mbp) [19]; **f)** qP-SC3.1-2019 (21.5-31.4 Mbp) [19]; **g)** qP-SC3.2-2019 (12.4-30.0) [19]; **h)** qP-FC3.2-2018 (14.5-33.7 Mbp) [19]; **i)** qP-FC3.1-2019 (21.5-33.8 Mbp) [19]; **j)** qP-FC3.2-2019 (13.5-32.8 Mbp) [19]; **k)** qP-FC3.2-2017 (18.3-24.9 Mbp) [19]; **l)** qP-FC3.1-2018 (21.5-32.9 Mbp) [19]

Notes: Several optical parameters for fruit skin and flesh colour were used in reference Sooriyapathirana *et* al. [17] resulting in overlapping QTLs. For mapping, the most distant markers of these QTLs summary were used.

Tab S4: SNP markers associated with firmness characters, phenotype mean values for genotypes, its analysis of variance, and mapping on QTLs

**Firmness of skin**												
						Phenotype mean values for alleles			Analysis of variance			
SNP	CHR	Position	P-value	MAF	Alleles	0/0	0/1	1/1	F	P-value	F critical	Mapping on QTLs
chr4_16000421	4	16,000,421	1.41E-25	0.25	T/A	5.66	5.99	7.39	219.54	3.80E-54	3.03	a, b
chr2_25034310	2	25,034,310	2.63E-17	0.14	T/A		6.95	6.72	3.07	8.13E-02	3.88	
chr1_31855038	1	31,855,038	9.66E-10	0.35	T/C	6.29	6.70	7.00	6.41	1.95E-03	3.03	c-e
chr7_25384148	7	25,384,148	1.17E-09	0.02	A/G		5.48	6.83	18.30	2.75E-05	3.88	
chr1_58902546	1	58,902,546	7.35E-09	0.14	C/A	5.17	6.13	7.03	30.07	2.48E-12	3.03	
**Firmness of flesh**												
						Phenotype mean values for alleles			Analysis of variance			
SNP	CHR	Position	P-value	MAF	Alleles	0/0	0/1	1/1	F	P-value	F critical	Mapping on QTLs
chr4_16000421	4	16,000,421	1.68E-25	0.25	T/A	4.52	5.12	6.91	184.72	1.21E-48	3.03	a, b
**Value of penetration**												
						Phenotype mean values for alleles			Analysis of variance			
SNP	CHR	Position	P-value	MAF	Alleles	0/0	0/1	1/1	F	P-value	F critical	Mapping on QTLs
chr8_19215607	8	19,215,607	6.92E-17	0.2	A/G		0.33	0.44	117.29	2.85E-22	3.88	
chr6_41860311	6	41,860,311	1.20E-13	0.06	A/G	0.44	0.44	0.39	2.60	7.67E-02	3.04	f
chr2_43495800	2	43,495,800	1.05E-12	0.46	C/A	0.35	0.39	0.45	19.21	2.00E-08	3.04	
chr6_18346767	6	18,346,767	1.14E-10	0.1	A/G		0.33	0.42	38.43	2.68E-09	3.88	g
chr1_14345045	1	14,345,045	1.11E-09	0.42	C/T	0.41	0.41	0.38	2.36	9.63E-02	3.04	
chr2_27724952	2	27,724,952	2.51E-09	0.1	A/C	0.56	0.40	0.40	4.91	8.18E-03	3.04	
chr6_13575852	6	13,575,852	3.36E-09	0.34	-/T	0.39	0.41	0.38	3.65	2.76E-02	3.04	g
chr3_19735173	3	19,735,173	6.29E-08	0.32	T/G	0.35	0.40	0.41	3.18	4.36E-02	3.04	

Fruit bruiseness												
						Phenotype mean values for alleles			Analysis of variance			
SNP	CHR	Position	P-value	MAF	Alleles	0/0	0/1	1/1	F	P-value	F critical	Mapping on QTLs
chr4_16229770	4	16,229,770	3.06E-37	0.23	A/G	5.24	5.71	7.32	142.39	5.10E-41	3.03	a, b
chr1_61827812	1	61,827,812	2.55E-21	0.15	C/A	4.61	6.07	6.88	24.65	1.99E-10	3.03	
chr1_6099740	1	6,099,740	3.81E-11	0.22	A/G	5.20	6.24	6.96	29.22	4.86E-12	3.03	
chr6_34665604	6	34,665,604	1.06E-09	0.09	T/C		6.08	6.74	12.94	3.94E-04	3.88	f
chr3_26046798	3	26,046,798	2.80E-09	0.37	G/A	6.17	6.63	6.74	2.77	6.46E-02	3.03	
chr7_25384148	7	25,384,148	4.61E-09	0.02	A/G		4.76	6.68	25.84	7.63E-07	3.88	

a) qP-FF4.1 (14.1-16.8 Mbp) [22]; **b)** qP-FF4.1^{m} (14.8-16.8 Mbp) [13]; **c)** qP-FF1.1^{m} Y1 (18.0-31.9 Mbp) [23]; **d)** qP-FF1.2^{m} Y1 (20.7-32.7 Mbp) [23]; **e)** qP-FF1.2^{m} (22.3-32.7 Mbp) [23]; **f)** qP-FF6.1^{m} (28.0 Mbp-end of chr6) [13]; **g)** qP-FF6.1^{m} Y2 (10.8-27.1 Mbp) [23]

Notes: Different publications used different methods for fruit firmness assessments, all previously known QTLs were used for SNP mapping regardless of their origin.

Tab. S5: SNP markers associated with size characters, phenotype mean values for genotypes, its analysis of variance, and mapping on QTLs

Fruit length												
						Phenotype mean values for alleles			Analysis of variance			
SNP	CHR	Position	P-value	MAF	Alleles	0/0	0/1	1/1	F	P-value	F critical	Mapping on QTLs
chr1_43682223	1	43,682,223	1.04E-18	0.13	A/T	20.48	21.83	21.08	3.44	3.39E-02	3.03	
chr2_29776624	2	29,776,624	2.88E-16	0.42	T/C	19.26	20.99	22.56	49.96	9.33E-19	3.03	a, b
chr8_28900983	8	28,900,983	1.15E-15	0.5	T/G	21.14	21.19	21.40	0.30	7.39E-01	3.03	
chr6_34823266	6	34,823,266	1.28E-11	0.18	G/T	16.92	20.40	21.75	27.14	2.60E-11	3.03	
chr8_27088406	8	27,088,406	1.04E-10	0.23	T/A	19.01	20.45	21.89	30.54	1.70E-12	3.03	
chr5_11497974	5	11,497,974	2.85E-10	0.01	G/A		22.69	21.19	4.11	4.39E-02	3.88	
chr2_29275642	2	29,275,642	3.40E-10	0.25	T/C	19.40	20.65	21.82	16.48	2.04E-07	3.03	a
chr4_5976608	4	5,976,608	2.35E-09	0.2	G/C	19.15	20.28	21.84	28.55	8.33E-12	3.03	
Fruit width												
						Phenotype mean values for alleles			Analysis of variance			
SNP	CHR	Position	P-value	MAF	Alleles	0/0	0/1	1/1	F	P-value	F critical	Mapping on QTLs
chr2_31850964	2	31,850,964	7.94E-32	0.38	G/A	19.25	22.28	23.98	84.66	2.62E-28	3.03	a-d
chr1_33914270	1	33,914,270	4.01E-15	0.15	T/C	20.26	20.85	23.18	36.56	1.61E-14	3.03	e,f
chr5_24153701	5	24,153,701	1.78E-13	0.14	C/T	20.21	21.17	22.99	22.81	9.10E-10	3.03	
chr8_14165946	8	14,165,946	1.09E-09	0.02	G/T		21.64	22.56	1.38	2.42E-01	3.88	
chr3_18843170	3	18,843,170	4.88E-09	0.02	G/C		23.98	22.46	4.68	3.15E-02	3.88	
chr4_16680250	4	16,680,250	5.27E-09	0.03	A/G	16.11	20.17	22.67	15.48	4.91E-07	3.03	
chr8_19751357	8	19,751,357	1.26E-08	0.32	T/A	21.92	22.71	22.49	1.32	2.69E-01	3.03	
chr2_7960492	2	7,960,492	2.30E-08	0.02	C/A		21.45	22.57	2.29	1.31E-01	3.88	

Fruit thickness												
						Phenotype mean values for alleles			Analysis of variance			
SNP	CHR	Position	P-value	MAF	Alleles	0/0	0/1	1/1	F	P-value	F critical	Mapping on QTLs
chr2_29776725	2	29,776,725	2.53E-13	0.42	G/A	17.55	19.75	21.11	84.72	2.54E-28	3.03	a, b
chr5_24274628	5	24,274,628	2.10E-10	0.13	A/C	16.02	19.03	20.15	21.62	2.48E-09	3.03	
chr5_23342227	5	23,342,227	2.70E-10	0.03	A/T	20.02	17.41	12.02	30.59	1.63E-12	3.03	
chr4_17924819	4	17,924,819	6.68E-09	0.09	G/A	15.06	18.45	20.17	34.44	8.13E-14	3.03	
chr4_26059601	4	26,059,601	1.34E-08	0.04	A/T		20.10	19.81	0.51	4.77E-01	3.88	
chr4_6228894	4	6,228,894	1.81E-08	0.11	G/T	18.35	18.86	20.10	12.08	1.02E-05	3.03	
**Weight of fruit**												
						Phenotype mean values for alleles			Analysis of variance			
SNP	CHR	Position	P-value	MAF	Alleles	0/0	0/1	1/1	F	P-value	F critical	Mapping on QTLs
chr2_29787028	2	29,787,028	1.11E-23	0.41	G/A	4.39	5.72	6.91	67.16	1.02E-23	3.03	a, b
chr6_16582783	6	16,582,783	3.94E-11	0.01	G/C		8.84	5.87	20.06	1.18E-05	3.88	
chr1_33914270	1	33,914,270	1.19E-09	0.15	T/C	4.71	4.88	6.32	34.11	1.05E-13	3.03	e,f
chr1_31819328	1	31,819,328	7.88E-09	0.39	C/A	4.93	6.13	5.89	8.65	2.38E-04	3.03	e-g
chr4_5976608	4	5,976,608	8.26E-09	0.2	G/C	4.36	5.30	6.34	27.64	1.73E-11	3.03	
chr1_24330881	1	24,330,881	1.20E-08	0.08	G/T		6.59	5.79	11.72	7.32E-04	3.88	e-h

a) Ma069a-MA005c (28.4-33.7 Mbp) [24]; **b)** qP-FW2.1^{m} (29.4-41.2 Mbp) [13]; **c)** QTL analyzed for haplotypes CPSCT038-BPPCT034 (30.3-33.3 Mbp) [24]; **d)** FW_G2a (30.3-33.3 Mbp) [25]; **e)** FW_G1 (24.3-37.6 Mbp) [25]; **f)** qP-FW1.1^{m} (16.3-39.8 Mbp) [13]; **g)** qP-FD1.1^{m} Y1 (21.0-33.8 Mbp) [23]; **h)** qP-FD1.1^{m} Y2 (15.0-31.5 Mbp) [23]

Notes: Different publications used different measures (i.e. diameter, length, size, and weight) for these closely linked fruit parameters, all previously known QTLs were used for SNP mapping regardless of their origin.

Tab. S6: Examples for fruit and flesh colour evaluation

Characteristics	Evaluation	Description	Examples
Fruit colour	1	yellow	Gold Sweet Cherry[74], Bigarreau d'Or[75], Dönnissens Gelbe[75]
	2	yellow with blush	Vega[75]
	3	yellow with face (marble)	Napoleonova[74], Tardif de Vignola[75]
	4	light red	Krupnoplodnaya[75]
	5	red	Montmorency[74], Alex[75], Sunburst[75]
	6	brown red	Burlat[75], Kordia[75], Lapins[75]
	7	dark red	Karesova[74], Hedelfinger Riesenkirsche[75], Stella[75]
	8	blackish	Annabella[75], Namosa[75]
	9	black	Knauffs Schwarze[74]
Flesh colour	1	yellowish white	Kralovna Hortensie[74]
	2	yellow	Dönnissens Gelbe[75]
	3	yellow	Napoleonova[74]
	4	pink	Reverchon[75], Sunburst[75]
	5	red	Germersdorfi 45[75], Hedelfinger Riesenkirsche[75]
	7	dark red	Karesova[74], Rubin[75], Szomolyai fekete[75]
	9	black red	Vladimirskaja[74]

Table S7: Examples for firmness evaluation

Characteristics	Evaluation	Description	Examples
Firmness of skin	1	very soft	
	3	soft	Merpet
	5	intermediate	Rivan, Merton Heart, Merton Glory
	7	firm	Summit, Rainier, Sweetheart
	9	very firm	Bianca, Lambert, Giorgia, Droganova
Firmness of flesh	1	very soft	Kralovna Hortenzie[74]
	3	soft	Early Rivers[74, 75]
	5	intermediate	Toprichterova[74], Kordia[75], Sunburst[75]
	7	tight	Bigarreau cherries[74], Reverchon[75], Van[75]
	9	very tight	Moser[74], Kavics[75], Sumtare[75]
Fruit bruiseness	1	very high	Kralovna Hortenzie[74]
	3	high	
	5	intermediate	Karesova[74]
	7	low	Kordia[74]
	9	absent	Moser[74]
